# Is Cognitive Training Effective for Improving Executive Functions in Preschoolers? A Systematic Review and Meta-Analysis

**DOI:** 10.3389/fpsyg.2019.02812

**Published:** 2020-01-10

**Authors:** Nicoletta Scionti, Marina Cavallero, Cristina Zogmaister, Gian Marco Marzocchi

**Affiliations:** Department of Psychology, University of Milan – Bicocca, Milan, Italy

**Keywords:** executive functions, EF training, cognitive training, preschoolers, meta-analysis

## Abstract

In the present meta-analysis, we examined the effect of cognitive training on the Executive Functions (EFs) of preschool children (age range: 3–6 years). We selected a final set of 32 studies from 27 papers with a total sample of 123 effect sizes. We found an overall effect of cognitive training for improving EF (*g* = 0.352; k = 123; *p* < 0.001), without significant difference between near and far transfer effects on executive domains. No significant additional outcome effects were found for behavioral- and learning-related outcomes. Cognitive training programs for preschoolers are significantly more effective for developmentally at-risk children (ADHD or low socio-economic status) than for children with typical development and without risks. Other significant moderators were: individual vs. group sessions and length of training. The number of sessions and computerized vs. non-computerized training were not significant moderators. This is the first demonstration of cognitive training for transfer effects among different executive processes. We discuss this result in relationship to the lower level of modularization of EFs in younger children.

**Protocol Registration**: PROSPERO (CRD42019124127). Available online at: https://www.crd.york.ac.uk/prospero/display_record.php?ID=CRD42019124127.

## Introduction

Executive Functions (EFs) are a set of top-down cognitive processes that underpin goal-directed behaviors (Shallice and Burgess, 1996; Diamond, 2013).

EFs have been distinguished in three major core executive processes: Working Memory (WM), Inhibitory Control (IC), and Cognitive Flexibility (CF) (Miyake et al., 2000). WM refers to holding in mind and mentally manipulating information. IC refers to the ability to resist impulses, distractions, and habits, and to actively suppress interfering representations for producing an adequate response. CF refers to the ability to *think outside the box* and adjust to change (Diamond, 2013). These skills allow us to monitor and flexibly adapt our behavior to changes in context, and to learn new actions and strategies to solve new and complex problems.

Over the past decades, an increasing body of empirical results has demonstrated that the development of EFs during early childhood plays an important role in supporting school readiness and social-emotional development, and in predicting which cognitive abilities will be required for succeeding in school (Best et al., 2011). Furthermore, EFs are also critical cognitive domains for understanding the heterogeneous nature of neurodevelopmental disorder phenotypes, since EFs impairments have already been observed at this age in neurodevelopmental disorders, such as Attention Deficit Hyperactivity Disorder (ADHD), Autism Spectrum Disorder (ASD), and Specific Language Impairment (SLI) (Craig et al., 2016; Slot and Von Suchodoletz, 2018).

Preschool age marks the passage from infancy to childhood and represents the most critical period for child development (Diamond, 2006; Garon et al., 2008; Best and Miller, 2010). In this period of life, we experience major performance improvements in many EF tasks, in parallel with structural and functional changes of the prefrontal cortex, like the wide pruning of synaptic connections (Huttenlocher, 1979) and the maturation of subcortical prefrontal myelination (Kinney et al., 1988). The rapid changes occurring in preschoolers make it difficult to define the organization of EFs clearly. Contrary to adulthood, in which there is a general consensus about the EFs multi-domain structure, the question of the development and the structure of EFs in early childhood is still open. Studies have provided empirical evidence in support of both a global unitary nature and a multifaceted nature of the EFs structure over the preschool years, although both the number and the nature of these functions have differed across studies.

Given the rapid and heterogeneous nature of EFs development over the preschool period (Howard et al., 2015), the practice of investigating the issue by collapsing participants into overly large age bands might have obscured qualitative age-related differences in their organization. Furthermore, Howard et al. (2015) reported that EFs followed dynamic developmental trajectories every 6 months across the preschool period, and did not become linearly more differentiate, as was largely theorized by factorial studies. Moreover, Nelson et al. (2016) found that the degree of unity of EFs during these years did not decrease linearly over time.

Studies targeting the EFs structure in 3 year old children found that a unitary model describes the EFs organization better than a two- or three-factor structure (Hughes et al., 2010; Wiebe et al., 2011), while those focusing exclusively on children of 4, 5, and 6 years have found both a two-factor (Usai et al., 2014; Stålnacke et al., 2019) and a latent single-factor model (Wiebe et al., 2008; Fuhs and Day, 2011). Garon et al. (2008) proposed a hierarchical integrative model, in which each executive component is built on earlier developing functions in the first years of life, whose precursor is attention. Working memory is the component that develops first, followed by inhibitory control and, finally, cognitive flexibility is built on both of them. Diamond (2013) considers EFs as a unitary construct with three separable components, which develop supporting each other and all together carry out higher order executive processes (i.e., problem solving and planning, also referred as fluid intelligence).

Recent research comparing unitary vs. fractionated EFs models-fit in the entire preschool age band (Miller et al., 2012; Lerner and Lonigan, 2014; Howard et al., 2015; Monette et al., 2015), has highlighted methodological issues related to task selection in the studies supporting a single-domain organization of EF. This new evidence suggests that a two-factor structure comprising WM and IC as *diverse but united* components may summarize and better explain EFs during the preschool period.

Due to the important role of EFs for many aspects of human life (Best et al., 2011), many recent empirical studies have focused on cognitive training aimed at improving EFs and their precursors (e.g., attention) in preschoolers. The idea that EF impairments may place constraints on other higher cognitive functions suggests that, if training can enhance EFs, this should produce transfer effects to diverse tasks that place demands on the untrained executive processes and have important benefits for aspects of everyday functioning that are widely considered to depend on EFs.

These effects are commonly differentiated in near- and far-transfer effects. *Near-transfer effects* refer to the effects of cognitive interventions on various tasks tapping onto the same trained cognitive mechanisms (Melby-Lervåg and Hulme, 2013; Sala and Gobet, 2016, 2017; Kassai et al., 2019). *Far-transfer effects* refer to the effects of training on various aspects of behavior and learning, functionally related (but distinct) to Executive Functioning (Melby-Lervåg and Hulme, 2013; Sala and Gobet, 2016, 2017). However, some authors refer to far transfer effects also with reference to tasks tapping onto other executive processes, not directly trained by the intervention activities (Kassai et al., 2019), because it is questionable whether, in the case of children, training one EF has an effect on other, untrained, executive skills.

For instance, the near transfer effects of visuo-spatial working memory training in preschoolers would be measured on tasks such as Corsi backward or matrix span tasks, while far transfer effects of the same training in preschoolers would be measured on numeracy/literacy skills (learning) or Stroop-like tasks (inhibitory control, not directly trained).

Some studies demonstrated that children at risk (i.e., children from low-income families, with psychopathology traits, born preterm) may benefit particularly from EF programs, since improvement in EFs may lead to better academic performance (Diamond and Lee, 2011) and generally to better adaptation, leveling the playing field and reducing the achievement gap (St. John et al., 2019). Given the relevance of EFs in human life, it could be useful to also sustain and enhance the development of these skills in typically developing preschoolers. Empirical evidence showed that cognitive training may improve near-trained EFs across childhood, particularly for working memory (Wass et al., 2012), but to date there are no meta-analytic studies on their effectiveness focused in the 3–6 age range, which represents a critical period for EFs development.

Referring to cognitive interventions, Diamond and Lee (2011) found that training inhibitory control significantly improved these skills, but its effects do not generalize to delay of gratification performance in school-age children. As concerns working memory treatments, in a meta-analysis of 23 published training studies, Melby-Lervåg and Hulme (2013) concluded that these interventions led to reliable improvements in working memory skills, but the improvement did not transfer to other skills, such as reasoning, inhibitory processes, word decoding, and arithmetic skills. Still, Sala and Gobet (2017) reported only a small far-transfer effect of working memory training on mathematics and literacy in school-aged typically developing children, but no transfer to fluid intelligence. Recently, Kassai et al. (2019) and Takacs and Kassai (2019) reviewed the effectiveness of multi-domains and single-domain EFs training in 2–12 year old children, finding no convincing evidence of far-transfer among EFs themselves, or in multi-domain EFs cognitive training. Thus, although meta-analytic studies so far have shown that training EFs is possible, the transfer seems to be narrow and limited to tasks tapping the trained abilities.

However, the current meta-analysis specifically focuses on studies targeting only the preschool population. Since the EF components are still developing and less differentiated between the ages of 3–6 years than in middle childhood, cognitive training aimed at improving one or more of executive skills might also show significant effects on untrained EF tasks in this younger population. Furthermore, far-transfer effects may occur when children, during the training activities, learn and automatize a new cognitive routine, which is not yet established in their mind architecture (Gathercole et al., 2019). For instance, cognitive training tasks that load heavily on working memory skills might improve math performance; controlling, regulating, and actively maintaining relevant numerical information are fundamental processes to accomplish mental and written calculation, as well as number dictation and problem solving. As, plausibly, new cognitive routines are more easily established in preschoolers than in older children, we should therefore expect that far transfer effects to learning and behaviors are more likely in preschoolers. Many researches showed that targeting younger individuals have reported more widespread transfer of training effects and young children have generally shown significantly larger benefits from training than older children (Wass et al., 2012; Melby-Lervåg and Hulme, 2013).

Accordingly, our study aims to examine the evidence regarding the near- and far-transfer effects of cognitive training in preschoolers aged between 3 and 6 years old. To investigate these effects, we refer to Diamond's hierarchical model (Diamond, 2013), thus considering both core and high-level EF: working memory, inhibitory control, cognitive flexibility, planning, and problem solving (also referred to as fluid intelligence). Based on the literature, we also took into consideration that far transfer effects both behaviors and cognitive skills predicted or related to EFs development.

We included data of both typically developing and developmentally at-risk children, with the aim to contribute to the existing knowledge on the clinical question on the effectiveness of cognitive training for improvements in EFs and children's everyday functioning.

Based on the literature, the following hypothesis and research questions were investigated:

1. we expected significant near-transfer effects and possible significant far transfer effects of cognitive training both on the untrained executive components and on additional outcomes related to EFs, such as learning related processes (i.e., numeracy and literacy), adaptive and problem behaviors (e.g., inattention, hyperactivity, and impulsivity);
2. we also wanted to investigate if such transfer effects would vary across:

- targeted population: (a) age of participants, (b) developmentally at-risk children vs. not-at-risk children.
- type of control group: (c) active vs. passive
- characteristics of the training: (d) computerized vs. not computerized; (e) individual vs. group; (f) number of sessions; (g) length in minutes.

## Methods

### Operational Definitions

We categorized the cognitive interventions based on the characteristics of the training and on the EF it targeted. Specifically, we categorized the training as *computerized* when activities were carried out with the help of a computer, tablet, robot, or virtual reality and *not computerized* when they were conducted in a classical manner, that included paper and pencil tasks and/or activities involving the children's bodies. All interventions utilized game-like activities aimed at improving one or more EF skills by practicing tasks involving a precursor of EFs, such as attention, or one or more executive processes.

As reported by Takacs and Kassai (2019), the main feature of EFs training is that children are not given new strategies, but they have to apply their own existing set of strategies. We categorized training as *group* interventions when it was based on the presence of small groups of peers during the activities, and *individual* interventions, when based only on trainer-child interactions. To differentiate near- and far-transfer effects we categorized each outcome measure according to which major executive process it assessed, based on the preschool EFs assessment literature (Garon et al., 2008; McCormack and Atance, 2011; Anderson and Reidy, 2012; Diamond, 2013). For instance, tasks requiring active manipulation of information kept in mind, such as backward digit, word, or spatial span tasks, were coded respectively as verbal and visuospatial working memory measures, as well as those that involved mostly memory updating processes (e.g., keep track, Mr. X or Odd-One-Out). Forward span-like tests were considered to measure short-term memory since they did not require working memory processes (Alloway et al., 2006) therefore, we did not include them in the meta-analysis. If a study collapsed forward and backward trials in a single measure, we included it as a general measure of working memory process. We considered those tests that required children to inhibit either a distractor (Commissions of the Continuous Performance Test), a prepotent (Stroop like task and Go/NoGo paradigm), or automatized response (Head Toes Knees Shoulders), as well as tasks requiring them to wait for gratification, as measures of inhibitory control. We categorized tests requiring a *shift* among different response sets and flexibly adjusting the response according to new rules (e.g., last phase of Shape School, Trail Making Test, and Dimensional Change Card Sort) as measures of cognitive flexibility. We classified tests that required the children to order events mentally in advance (McCormack and Hanley, 2011), such as Tower-like tasks, as measures of planning abilities; we considered tasks that challenged thinking, demanding to abstract, reason and recognize visuo-spatial pattern, such as Raven Matrices and Cube Drawing, as measures of problem solving skills.

It should be pointed out that we considered each of these outcome domains as separate to differentiate near- from far-transfer effects on untrained EFs processes. For example, we considered the effect of IC training on an IC task as near-transfer, while we categorized the effect of IC training on working memory, cognitive flexibility, planning, or problem solving tasks as far-transfer. Since to date there is no consensus among scientists over the factorial organization of EFs in early childhood, it is necessary to previously establish if far transfer from separate executive functions is possible, before addressing questions about generalization to other far-aspects, such as learning and behavior. We considered these far-aspects as additional outcomes, that is, measures of the effects on fields related to (but different from) EFs. This definition of far-transfer is consistent with Thorndike and Woodworth's (1901) common element theory. We collected three type of non-EF far-transfer measures, based on previous literature. Specifically, we considered effects on EFs related problem behaviors, including inattention, hyperactivity, and conduct issues, measured by a parent and teacher rating scale; on learning related outcomes, measured by early numeracy and literacy tasks or mean grades (at kindergarten); and EFs related behaviors including emotional self-regulation abilities, and social and adaptive abilities connected to EFs.

### Search Strategy

In accordance with the PRISMA statement (Moher et al., 2009), we used a systematic search strategy to find the pertinent studies. Using different combinations of the terms “executive functions,” “training,” and “preschoolers” and their synonyms (see Appendix A for a sample of the detailed search string), we searched on PubMed, PsycInfo, Web of Science, Dialnet, ERIC, Redalyc, ProQuest Dissertations & Theses Global, Base de Datos de Tesis Doctorales (TESEO), e-thesis online service (EThOS), DART-Europe E-theses Portal, and the Biblioteca Nazionale Italiana Doctoral Thesis Repository to identify all potential journal articles and unpublished studies, as doctoral dissertations, that reported on the effects of cognitive training programs aimed at improving EFs in children aged 3–6 years old. We also published posts on Researchgate, Facebook, Linkedin, and Twitter, sent e-mails to Italian Psychological Associations and researchers in the field, to invite researchers to send us their unpublished works and inaccessible data on the topic. Despite our extensive research of the gray literature, we found only a small amount of unpublished studies. Preliminary analyses ruled out the presence of publication bias; the size of the EF training effects was bigger in the unpublished studies. Adopting a conservative approach, we excluded the gray literature from the principal analyses to avoid a possible source of bias, due to their low numerosity and atypically high EF effects. However, a parallel analysis conducted including these studies, reported in Supplementary Material, showed that the differences in results were negligible.

**Figure 1:**
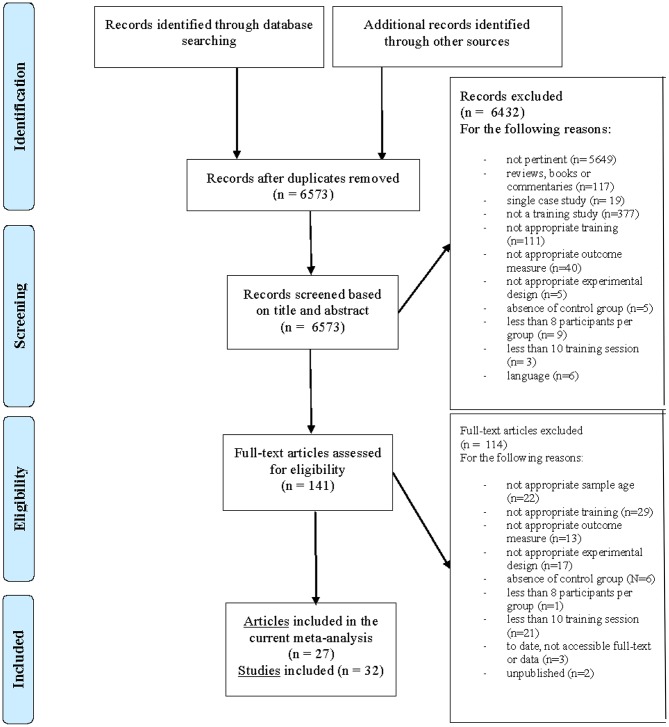
Prisma flow diagram.

After excluding duplicates, 6,573 records remained. The first and second authors independently screened all of them, based on title and abstract and according to inclusion and exclusion criteria. The agreement rate in this phase was 98%. As a secondary search, the references of the selected studies (*n* = 141), in addition to relevant systematic reviews, were checked to find other eligible studies. Full texts of the identified papers were reviewed by the first and second author and we solved disagreements through discussion with the fourth author. Also in this phase, the agreement rate between the two raters was high (95%). Finally, as shown in the flow chart, we identified 27 articles (32 studies) with 123 contrasts that were eligible for the present meta-analytic review. Details concerning the method of literature search and criteria for inclusion and exclusion of studies are shown in Figure 1.

### Inclusion Criteria

The included studies had to meet the following criteria:

- at least one EF outcome measure;
- pre-post treatment designs and randomized control trials with at least a (either active or passive) control group;
- at least one of the EF measures was an objective neurocognitive measure;
- at least 8 participants per condition;
- at least 10 sessions;
- paper written in English, Italian or Spanish.

### Exclusion Criteria

Firstly, we excluded all the studies where participants were not 3–6 years old. In doing so, we strictly considered the age criterion (3–6 years old children), disregarding grade attendance since preschool attendance years vary across countries and we could have potential review papers from about 63 different countries (calculate based on Lewis's *Ethnologue Language of the World* data; Lewis, 2009). This decision was also substantiated by evidence over the 5–6 years period, which revealed there are no relevant differences among EFs organization between older preschoolers and first graders (Usai et al., 2014).

Then, we excluded all studies utilizing training strictly based on physical exercise, drama, and art activity, as well as preschool curricula created to enhance EFs, mindfulness-based, and neurofeedback training, because the current meta-analysis aims to establish the effect of cognitive training to EFs. We also excluded works that combined cognitive training with parent training or, more generally, were part of a multimodal system intervention, because we are interested in disentangling the effects of cognitive training from other types of intervention in combination.

With regards to outcome measures, we included performance-based measures collected by EFs tasks and EFs-related cognitive abilities, like literacy, numeracy, and academic achievement. All outcomes were based on continuous data. To avoid near-transfer overestimation, we excluded from the analysis outcome measures that were merely based on the same tasks practiced during the intervention. Furthermore, we run additional analyses on far transfer combining verbal and visuo-spatial working memory (WM) into a single outcome (see Supplementary Material) to avoid executive far-transfer overestimation, since the two dimensions of WM could be more related to each other than other analyzed processes. Where available, instead of reaction time, we reported accuracy or error rates, due to their higher reliability across childhood (Diamond et al., 2007). We included only studies having at least 8 children per group, as smaller sample sizes would increase the risk of publication bias. We included only studies having at least 10 training sessions, as from a clinical point of view, this number can be considered the minimum amount of sessions required to observe improvement in the EFs development.

Finally, we accepted measures of problem behaviors and social-emotional aspects collected through teacher and parent reports, but only if studies also reported at least one neurocognitive EFs measure.

### Coding

During the coding phase, the first and second author coded each record according to a predefined coding schema, collecting information about bibliographic information [i.e., title, author(s), and year of publication], sample characteristics (i.e., sample size, mean age, and standard deviation of each group, clinical risk status of the sample), characteristics of the cognitive training (i.e., individual vs. group and computerized vs. non-computerized), its duration in term of number of sessions and total duration in minutes, type of control group involved (i.e., active or passive), the kind of outcome measure (i.e., verbal and visuo-spatial working memory, inhibitory control, cognitive flexibility, planning, and problem solving), and additional outcome measures (i.e., learning, behaviors, and problem behaviors related constructs), the near and far transfer measures.

For the studies reporting more than one intervention or control condition that met our inclusion criteria, we included more contrasts. If there were two or more eligible cognitive training programs, these were both included as compared with the control group (Thorell et al., 2009; Bergman Nutley et al., 2011; Howard et al., 2017; Romero López, 2018; Zhang et al., 2018). We did the same when there were multiple control conditions like an active and a passive control in the same study (i.e., Thorell et al., 2009; Peng et al., 2017; Pozuelos et al., 2019). Furthermore, if there were two or more experimental conditions, we selected those that met the inclusion criteria as experimental conditions, while considering those that did not as active control conditions. For instance, Passolunghi and Costa (2016) tested the effects of working memory and early numeracy training alone by comparing them to a passive control condition. In this case, only working memory training met our inclusion criteria; thus we considered the early training condition an active control condition and compared both it and passive control group to WM training group (see also Kassai et al., 2019).

### Meta-Analytic Procedures

We used R version 3.5.1 (R Core Team, 2018), RStudio version 1.1.453 (R Studio Team, 2016), and the Metafor package (Viechtbauer, 2015; see Assink and Wibbelink, 2016) to conduct the analyses. R code and data are openly available as Supplementary Material.

We computed the size of the EFs effect as the standardized mean of the difference in the pre-post outcome change between the experimental and control group. We chose Hedges' *g* over Cohen's *d* because it corrects for small sample sizes (Borenstein et al., 2009). A positive *g*-value reflected the advantage of the intervention condition, while a negative effect indicated that the control group outperformed the intervention group. We computed Hedge's *g* based on Morris (2008). The summary statistics required for each outcome were the number of participants in intervention and control groups, the mean value of the outcomes in each group pre and post-treatment (or, as an alternative, the mean change from baseline), and the pooled pre-intervention standard deviation. For one study (Traverso et al., 2015), the available data did not allow to compute the effect size following Morris (2008). However, the authors reported the Cohen's *d*, and we computed *g* based on this value.

As discussed before, many studies in the dataset reported several potentially correlated relevant outcomes, and some studies comprised multiple control groups or multiple intervention groups, which caused the same group to be present in more than one contrast. Both of these aspects created dependencies in the data. So far, several solutions have been introduced to avoid dependency (Borenstein et al., 2009; Assink and Wibbelink, 2016): analyzing the outcomes as if they were independent (i.e., ignoring the dependency), averaging the dependent outcomes into a single effect size, selecting only one outcome for each study, and multilevel meta-analysis. Ignoring the dependency might bias the results; averaging or eliminating effect sizes, on the other hand, would decrease the power of the analysis and limit the research questions that we could ask, as we would not be able to compare near and far transfer effects. We, therefore, conducted a three-level meta-analytic analysis, following Assink and Wibbelink (2016). The meta-analytic model considered three different sources of variance: the participants at level 1, the outcomes at level 2, and the studies at level 3.

We used the rma.mv function of the Metafor package and set the tdist parameter as TRUE. Therefore, we based the test statistics and confidence intervals on the t distribution, applied the Knapp and Hartung (2003) adjustment, and used the Restricted Maximum Likelihood estimation method (REML) for estimating the parameters.

## Results

### Included Studies

Thirty-two studies were eligible for inclusion, for a total of 123 different outcomes, with 977 participants in the training, 341 participants in the active control, and 719 in the passive control conditions.

Tables 1, 2 summarized the characteristics of the studies: in particular, in Table 1, EF measures and near-far transfer effects are reported; in Table 2, we described additional outcome measures.

**Table 1:** Summary of the studies included into the meta-analysis: EF outcome measures and Near vs. Far Transfer effects.

**References**	**Mean age (in months)**	**Clinical risk status of sample**	**Training condition**	**Number of session**	**Control condition**	**Executive outcome measure**	**Type of transfer**	**Hedge's g, [95% CI]**
Bergman Nutley et al., 2011	51,2	Typically developing	Visuospatial WM training (Individual, Computerized)	25	Passive control (*n* = 25)	Fluid intelligence		
Contrast 1			(*n* = 24)			- Raven matrix - Block design	Far transfer	−0.164, [−0.320, −0.008]
						- Leiter's problem Solving task	Far transfer	−0.118, [−0.273, 0.037]
						Visuospatial WM		
						- Odd one out	Near transfer	0.889, [0.718, 1.060]
						Verbal WM		
						- Word span	Far transfer	0.216, [0.060, 0.372]
Bergman Nutley et al., 2011	51,2	Typically developing	Non-verbal reasoning training (Individual, Computerized)	25	Passive control (*n* = 25)	Fluid intelligence		
Contrast 2			(*n* = 24)			- Raven matrix - Block design	Near transfer	0.302, [0.146, 0.459]
						Visuospatial WM		
						- Verbal dual task	Far transfer	0.295, [0.138, 0.452]
						- Odd one out	Far transfer	0.553, [0.392, 0.714]
						Verbal WM		
						- Word span	Far transfer	−0.068 [−0.223, 0.087]
Bergman Nutley et al., 2011	51,2	Typically developing	Combined visuospatial WM and Non-verbal reasoning training (Individual Computerized)	25	Passive control (*n* = 25)	Fluid intelligence		
Contrast 3			(*n* = 27)			- Raven matrix - Block design	Near transfer	0.350, [0.202, 0.499]
						Visuospatial WM		
						- Odd one out	Near transfer	0.775, [0.617, 0.933]
						Verbal WM		
						- Word span	Far transfer	−0.019, [−0.165, 0.128]
Brock et al., 2018	72,84	Typically developing	Executive functions training (Group)	73	Active control (*n* = 43)	Inhibitory control and cognitive flexility	
			(*n* = 44)			- Nepsy (three subtests)	Near transfer	0.721, [0.627, 0.815]
Capodieci et al., 2018	65,88	ADHD symptoms	Working Memory training Sviluppare la concentrazione e l'autoregolazione. Giochi e attività sul controllo della memoria di lavoro (both Individual and Group)	16	Passive control (*n* = 16)	Verbal WM		
			(*n* = 18)			- Backward digit span	Near Transfer	1.171, [0.911, 1.431]
						Visuospatial WM		
						- Selective working memory	Near Transfer	0.719, [0.483, 0.954]
						Inhibitory control		
						- Matching familiar figures	Far transfer	0.464, [0.237, 0.691]
						- Walk nowalk	Far transfer	0.717, [0.481, 0.952]
								
Foy and Mann, 2014	62,15	Low SES	Visuo-spatial WM training Cogmed JM (Individual, Computerized)	25	Passive control (*n* = 28)	Visuospatial WM		
			(*n* = 23)			- Corsi backward	Near transfer	0.465, [0.310, 0.619]
						Verbal WM		
						- Digit backward	Far transfer	0.344, [0.191, 0.496]
						Inhibitory control		
						- HTKS	Far transfer	0.288, [0.136, 0.441]
Gade et al., 2017	62,39	Typically developing	Visuo-spatial WM training (Individual)	11	Active control (*n* = 10)	Verbal WM		
Study 1			(*n* = 10)			- Word span	Far transfer	0.230, [−0.132, 0.592]
						Visuospatial WM		
						- Matrix span	Near transfer	0.225, [−0.137, 0.587]
						- Object span task	Near transfer	0.361, [−0.005, 0.727]
Gade et al., 2017	67,19	Typically developing	Visuo-spatial WM training (Individual)	12,5	Active control (*n* = 16)	Verbal WM		
Study 2			(*n* = 15)			- Word span	Far transfer	0.643, [0.390, 0.896]
						Visuospatial WM		
						- Matrix span	Near transfer	0.108, [−0.133, 0.348]
						- Color span backward	Near transfer	−0.364, [−0.609, −0.120]
Gade et al., 2017	72	Typically developing	Visuo-spatial WM training (Individual)	13,5	Active control (*n* = 10)	Verbal WM		
Study 3			(*n* = 10)			- Word span	Far transfer	0.257, [−0.106, 0.619]
						Visuospatial WM		
						- Matrix span	Near transfer	−0.319, [−0.683, 0.046]
						- Color span backward	Near transfer	0.368, [0.002, 0.735]
Gade et al., 2017	61,3	Typically developing	Visuo-spatial WM training (Individual)	12	Active control (*n* = 10)	Verbal WM		
Study 4			(*n* = 10)			- Word span	Far transfer	−0.463, [−0.833, −0.093]
						Visuospatial WM		
						- Matrix span	Near transfer	−0.875, [−1.273, −0.478]
						- Color span backward	Near transfer	−0.574, [−0.950, −0.198]
Garcia Fernandez et al., 2018	74,39	Typically developing	Motor and executive functions training motor area Activity with executive functions program (Group)	45	Passive Control (*n* = 31)	Cognitive flexibility		
			(*n* = 35)			- Design fluency test	Near transfer	0.844, [0.717, 0.971]
						Inhibitory control		
						Inhibitory executive function test	Near transfer	0.255, [0.137, 0.372]
Howard et al. (2017)	52,79	Typically developing	Executive functions training using quincey Quokka's quest (Group)	10	Passive control (*n* = 18)	Visuospatial WM		
Contrast 1			(*n* = 22)			- MrAnt	Near transfer	0.205, [0.014, 0.397]
						Inhibitory control		
						- Go/No-Go	Near transfer	−0.046, [−0.237, 0.144]
						Flexibility		
						- Card sorting	Near transfer	0.415, [0.221, 0.609]
Howard et al., 2017	52,79	Typically developing	Executive functions training using Quincey Quokka's quest (Group)	10	Passive control (*n* = 18)	Visuospatial WM		
Contrast 2			(*n* = 25)			- MrAnt	Near transfer	−0.038, [−0.219, 0.142]
						Inhibitory control		
						- Go/No-Go	Near transfer	−0,185, [−0.367, −0.004]
						Flexibility		
						- Card sorting	Near transfer	0.851, [0.654, 1.048]
Joekar et al., 2017	68,8	ADHD symptoms	Visual attention training pay attention program (Individual)	11	Passive control (*n* = 15)	Inhibitory control		
			(*n* = 15)			- Toulouse pieron test (commission errors)	Far transfer	1.054, [0.771, 1.338]
Liu et al., 2015	58,61	Typically developing	Inhibitory control training (Individual, Computerized)	12	Active control (*n* = 20)	Inhibitory control		
			(*n* = 16)			- Stroop	Near transfer	0.129, [−0.083, 0.340]
						Verbal WM		
						- Backward digit span	Far transfer	0.060, [−0.151, 0.271]
						Fluid intelligence		
						- Raven matrix	Far transfer	0.632, [0.410, 0.854]
Romero López (2018)	67,19	Typically developing	Executive functions training EFE - 5 (Group)	21	Active control (*n* = 50)	Inhibitory control		
(Dissertation) Contrast 1			(*n* = 66)			- Luria's test	Near transfer	1.652, [1.561, 1.743]
Romero López (2018)	67,19	Typically developing	Executive functions training EFE - 5 Cog (Group)	21	Active control (*n* = 50)	Inhibitory control		
(Dissertation) Contrast 2			(*n* = 69)			- Luria's test	Near transfer	1.659, [1.569, 1.748]
Mulvey et al. (2018)	61,67	Low SES	Executive functions and motor training SKIP (Group)	12	Passive control (*n* = 57)	Inhibitory control		
			(*n* = 50)			- Head toes knee skip	Near transfer	0.477, [0.403, 0.552]
Passolunghi and Costa (2016)	65,1	Typically developing	WM training (Group)	10	Passive control (*n* = 18)	Verbal WM		
Contrast 1			(*n* = 15)			- Verbal dual task	Near transfer	0.968, [0.712, 1.224]
						Visuospatial WM		
						- Visuospatial dual task	Near transfer	0.990, [0.733, 1.247]
Passolunghi and Costa (2016)	65,235	Typically developing	WM training (Group)	10	Active control (*n* = 15)	Verbal WM		
Contrast 2			(*n* = 15)			- Verbal dual task	Near transfer	0.896, [0.622, 1.170]
						Visuospatial WM		
						- Visuospatial dual task	Near transfer	0.466, [0.212, 0.721]
Pellizzoni et al. (2019)	65,1	Typically developing	Executive functions training (Group)	20	Passive control (*n* = 51)	Inhibitory control		
			(*n* = 55)			- Delay (Time)	Near transfer	0.557, [0.481, 0.633]
						- Gift wrap (time)	Near transfer	0.327, [0.253, 0.401]
						- Gift wrap (violations)	Near transfer	0.203, [0.129, 0.276]
						- Circle drawing (Time)	Near transfer	0.496, [0.421, 0.572]
						- Day/Night	Near transfer	0.449, [0.374, 0.524]
						Verbal WM		
						- Backward word span	Near transfer	0, [−0.073, 0.073]
Peng et al. (2017)	58,67	Typically developing	WM training (Individual, Computerized)	14	Active control (*n* = 25)	Fluid intelligence		
Contrast 1			(*n* = 23)			- Raven matrix	Far transfer	1.144, [0.959, 1.330]
Peng et al. (2017)	58,91	Typically developing	WM training (Individual, Computerized)	14	Passive Control (*n* = 26)	Fluid intelligence		
Contrast 2			(*n* = 23)			- Raven matrix	Far transfer	0.743, [0.577, 0.910]
Pozuelos et al. (2019)	63,55	Typically developing	Executive attention training with metacognitive scaffolding (Individual Computerized)	10	Active control (*n* = 33)	Inhibitory control		
Contrast 1			(*n* = 33)			- Simon says	Near transfer	0.173, [0.056, 0.289]
						Verbal WM		
						- Backward digit span WISC	Far transfer	0.164, [0.047, 0.280]
						Fluid intelligence		
						- k-BIT matrix	Far transfer	0.610, [0.489, 0.732]
Pozuelos et al., 2019	63,6	Typically developing	Executive attention training (Individual, Computerized)	10	Active control (*n* = 33)	Inhibitory control		
Contrast 2			(*n* = 31)			- Simon says	Near transfer	0.318, [0.196, 0.439]
						Verbal WM		
						- Backward digit span WISC	Far transfer	1,73E-16, [−0.120, 0.120]
						Fluid intelligence		
						- k-BIT matrix	Far transfer	0.469, [0.346, 0.592]
Re et al., 2015	63,225	ADHD symptoms	Executive functions training Sviluppare la concentrazione e l'autoregolazione (Group)	17	Passive control (*n* = 13)	Inhibitory control		
Study 1			(*n* = 13)			- Walk no walk	Near transfer	0.263, [−0.023, 0.548]
Re et al., 2015	65,38	Typically developing	Executive functions training Sviluppare la concentrazione e l'autoregolazione (Group)	17	Passive control (*n* = 13)	Inhibitory control		
Study 2			(*n* = 13)			- Walk no walk	Near transfer	0.804, [0.497, 1.111]
Ríos et al., 2014	63,71	Typically developing	Planning training Prototipo online de entrenamiento Cognitivo (Individual, Computerized)	12	Passive control (*n* = 8)	Planning		
			(*n* = 8)			- Tower of Mexico	Near transfer	0.061, [−0.377, 0.499]
Rojas-Barahona et al., 2015	52,3	Low SES	WM training (Individual, Computerized)	16	Active control (*n* = 124)	Verbal and visuospatial WM		
			(*n* = 144)			- Visuospatial and phonological WM	Near transfer	0.551, [0.521, 0.581]
Röthlisberger et al., 2012	60,45	Typically developing	Executive functions training (Group)	30	Passive control (*n* = 38)	Inhibitory control		
Study 1			(*n* = 33)			- Simple flanker	Near transfer	0.060, [−0.048, 0.169]
						Flexibility		
						- Mixed flanker	Near transfer	0.513, [0.401, 0.626]
						Visuospatial WM		
						- Complex SPAN TASK	Near transfer	0.747, [0.631, 0.863]
Röthlisberger et al., 2012	72,9	Typically developing	Executive functions training (Group)	30	Passive control (*n* = 34)	Inhibitory control		
Study 2			(*n* = 30)			- Simple flanker	Near transfer	0.227, [0.107, 0.348]
						Flexibility		
						- Mixed flanker	Near transfer	0.335, [0.214, 0.457]
						Visuospatial WM		
						- Complex span task	Near transfer	0.291, [0.170, 0.412]
Rueda et al., 2012	64,7	Typically developing	Executive attention training (Individual, Computerized)	10	Passive control (*n* = 18)	Inhibitory control		
			(*n* = 19)			- ANT (commissions)	Near transfer	−0.153, [−0.357, 0.050]
						- ANT (executive task)	Near transfer	0.004, [−0.199, 0.207]
						- Delay of self gratification	Near transfer	0.459, [0.251, 0.668]
						Fluid intelligence		
						- k-BIT matrix	Far transfer	0.097, [−0.106, 0.300]
						Self regulation		
						- Gambling task	Far transfer	0.278, [0.073, 0.483]
Salvaguardia et al., 2009	71	ADHD symptoms	WM training Sviluppare la concentrazione e l'autoregolazione: Giochi e attività sul controllo della memoria di lavoro (Group)	21	Passive control (*n* = 14)	Visuospatial WM		
			(*n* = 18)			- Dual request selective task	Near transfer	0.613, [0.365, 0.861]
Schmitt et al., 2018	55,2	Typically developing	Executive functions training using block play (Individual)	14	Passive control (*n* = 35)	Inhibitory Control		
			(*n* = 24)			- Stroop	Near transfer	0.291, [0.155, 0.426]
						- Head Toes Knee Skip	Near transfer	0.153, [0.019, 0.288]
						Flexibility		
						- Card sorting	Near transfer	−0.019, [−0.153, 0.115]
Schmitt et al., 2015	51,645	Low SES	Executive functions training (Group)	16	Passive control (*n* = 150)	Inhibitory control		
			(*n* = 126)			- Head toes knee skip	Near Transfer	0.426, [0.397, 0.455]
						Flexibility		
						- Card sorting	Near Transfer	0.159, [0.130, 0.187]
Sivó Romero, 2016	58,5	Typically developing	WM training (both Individual and Group)	117	Passive control (*n* = 48)	Verbal WM		
(Dissertation)			(*n* = 49)			- Backward digit span	Near transfer	1.134, [1.042, 1.228]
						Inhibitory control		
						- Shape school (II)	Far transfer	−0.224, [−0.305, −0.144]
						Flexibility		
						- Schape school (III)	Far transfer	0.149, [0.069, 0.229]
Thorell et al., 2009	56	Typically developing	Visuospatial WM training Cogmed (Individual, Computerized)	25	Active Control (*n* = 14)	Visuospatial WM		
Contrast 1			(*n* = 17)			- Span board	Near transfer	0.442, [0.194, 0.691]
						Verbal WM		
						- Word spans	Far transfer	1.104, [0.823, 1.385]
						Inhibitory control		
						- Stroop	Far transfer	0.244, [0.0002, 0.488]
						- Go/No-Go	Far transfer	0.036, [−0.206, 0.278]
						Problem solving		
						- Block design	Far transfer	−0.026, [−0.268, 0.216]
Thorell et al., 2009	57	Typically developing	Visuospatial WM training Cogmed (Individual, Computerized)	25	Passive control (*n* = 16)	Visuospatial WM		
Contrast 2			(*n* = 17)			- Span board	Near transfer	0.699, [0.459, 0.940]
						Verbal WM		
						- Word spans	Far transfer	1.070, [0.809, 1.330]
						Inhibitory control		
						- Stroop	Far transfer	0.342, [0.112, 0.571]
						- Go/No-Go	Far transfer	−0.022, [−0.248, 0.204]
						Problem solving		
						- Block design	Far transfer	0.332, [0.102, 0.561]
Thorell et al., 2009	56	Typically developing	Inhibition training Cogmed (Individual, Computerized)	25	Active control (*n* = 14)	Visuospatial WM		
Contrast 3			(*n* = 18)			- Span board	Far transfer	−0.671, [−0.922, −0.421]
						Verbal WM		
						- Word spans	Far transfer	0.178, [−0.060, 0.415]
						Inhibitory control		
						- Stroop	Near transfer	0.208, [−0.029, 0.446]
						- Go/No-Go	Near transfer	−0.195, [−0.432, 0.043]
						Problem solving		
						- Block design	Far transfer	−0.060, [−0.297, 0.176]
Thorell et al., 2009	57	Typically developing	Inhibition training Cogmed (Individual, Computerized)	25	Passive control (*n* = 16)	Visuospatial WM		
Contrast 4			(*n* = 18)			- Span board	Far transfer	−0.414, [−0.639, −0.188]
						Verbal WM		
						- Word spans	Far transfer	0.178, [−0.044, 0.399]
						Inhibitory control		
						- Stroop	Near transfer	0.268, [0.045, 0.491]
						- Go/No-Go	Near transfer	−0.267, [−0.490, −0.045]
						Problem solving		
						- Block design	Far transfer	0.382, [0.158, 0.607]
Tominey and McClelland, 2011	54,5	Typically developing	Executive functions training red light purple light (Group)	16	Passive Control (*n* = 37)	Inhibitory control		
			(*n* = 28)			- Head toes knee skip	Near transfer	0.153, [0.033, 0.273]
Traverso et al., 2015	68,65	Typically developing	Executive functions training (Group)	12	Passive control (*n* = 32)	Inhibitory control		
			(*n* = 43)			- Delay time	Near transfer	0.693, [0.582, 0.804]
						- Gift wrap	Near transfer	0.435, [0.328, 0.543]
						- Circle drawing	Near transfer	0.346, [0.240, 0.453]
						- Matching familiar figures	Near transfer	0.445, [0.338, 0.553]
						- Arrow flanker	Near transfer	0.277, [0.171, 0.383]
						- Go/No-Go	Near transfer	−0.020, [−0.124, 0.085]
						- Dots	Near transfer	0.524, [0.416, 0.633]
						Verbal WM		
						- Backward digit span	Far transfer	0.426, [0.319, 0.533]
						Visuospatial WM		
						- Mr. Cucumber	Near transfer	0.267, [0.162, 0.373]
						- Keep track	Near transfer	0.643, [0.533, 0.753]
Volckaert and Noël, 2015	60,32	Typically developing	Inhibition training (Group)	16	Active control (*n* = 23)	Inhibitory control		
			(*n* = 24)			- Traffic Lights, Cat/Dog, Head Toes Knee Skip, Stroop	Near transfer	0.463, [0.297, 0.629]
						WM		
						- Catego span, Word span, Block tapping	Far transfer	0.733, [0.560, 0.905]
						Flexibility		
						- Traffick light, Cat/Dog, Monster (mixed condition)	Far transfer	0.362, [0.198, 0.526]
Zhang et al., 2018	73,5	Typically developing	Visuospatial WM training (Individual, Computerized)	20	Passive control (*n* = 22)	Verbal WM		
Contrast 1			(*n* = 20)			- Backward digit span	Far transfer	−0.215, [−0.396, −0.034]
						Inhibitory control		
						- AX CPT	Far transfer	−0.127, [−0.307, 0.054]
						Fluid intelligence		
						- Raven Matrix	Far transfer	0.192, [0.011, 0.373]
Zhang et al., 2018	73,5	Typically developing	Inhibitory control training (Individual, Computerized)	20	Passive control (*n* = 22)	Verbal WM		
Contrast 2			(*n* = 21)			- Backward digit span	Far transfer	0.198, [0.022, 0.375]
						Inhibitory control		
						- AX CPT	Near Transfer	−0.456, [−0.636, −0.275]
						Fluid intelligence		
						- Raven matrix	Far transfer	−0.057, [−0.232, 0.119]

**Table 2:** Summary of the studies included into the Meta-analysis: *Addition*al Outcome Measures.

**References**	**Age (in months)**	**Clinical status of the sample**	**Training condition**	**Control condition**	**Additional outcomes**	**Hedge's g, [95% CI]**
Brock et al., 2018	72,84	Typically developing	Executive functions training (Group) (*n* = 44)	Active control (*n* = 43)	Problem behaviors: Social Skills Improvement System + Child Behavior Rating Scale	−0.303, [−0.392, −0.213]
					Learning-related behaviors: Social Skills Improvement System + Child Behavior Rating Scale	−0.031, [−0.120, 0.057]
Capodieci et al., 2018	65,88	ADHD symptoms	Working Memory training *Sviluppare la concentrazione e l'autoregolazione. Giochi e attività sul controllo della memoria di lavoro* (both Individual and Group) (*n* = 18)	Passive control (*n* = 16)	Inattention: PDDAI (Identificazione Precoce del Disturbo da Deficit di Attenzione/iperattività per Insegnanti) (Teacher)	0.022, [−0.199, 0.243]
					Hyperactivity: IPDDAI (Teacher)	0.029, [−0.192, 0.249]
					WM items of IPDDAI (Teacher)	0.230, [0.008, 0.452]
					Inattention: IPDDAG (Identificazione Precoce del Disturbo da Deficit di Attenzione/iperattività per Genitori) (Parent)	−0.237, [−0.459, −0.015]
					Hyperactivity: IPDDAG (Parent)	0.177, [-0.044, 0.399]
Foy and Mann, 2014	62, 15	Low SES	Visuo-spatial WM training *Cogmed JM* (Individual, Computerized) (*n* = 23)	Passive control (*n* = 28)	Letter Knowledge: Letter Naming Fluency (LNF) subtest of the Dynamic Indicators of Basic Early Literacy Skills (DIBELSNext) assessment tool	1.191, [1.013, 1.368]
Garcia Fernandez et al., 2018	74,39	Typically developing	Motor and executive functions training *Motor area Activity with Executive Functions Program* (Group) (*n* = 35)	Passive control (*n* = 31)	Phoneme Awareness: First Sounds Fluency (FSF) subtest of the DIBELSNext test	−0.836, [−0.999, −0.672]
					Literacy: CUMANIN (Infant neuropsychological maturity questionnaire)—reading subscale	0.163, [0.046, 0.280]
					Literacy: CUMANIN (Infant neuropsychological maturity questionnaire)—writing subscale	0.482, [0.362, 0.601]
					Math: TEMA-−3 (Test of early mathematics ability-3)	0.212, [0.095, 0.329]
Joekar et al., 2017	68,8	ADHD symptoms	Visual Attention Training *Pay Attention Program* (Individual) (*n* = 15)	Passive control (*n* = 15)	Inattention: CSI-4 (Child symptom inventory-4) (Parent)	−0.083, [−0.330, 0.165]
					Inattention: CSI-4 (Teacher)	−0.209, [−0.457, 0.040]
					Hyperactivity: CSI-4 (Parent)	0.461, [0.207, 0.716]
					Hyperactivity: CSI-4 (Teacher)	0.441, [0.187, 0.695]
Romero López (2018) (Dissertation) Contrast 1	67,19	Typically developing	Executive functions training *EFE – 5* (Group) (*n* = 66)	Active control (*n* = 50)	EF: BRIEF-P (Behavior rating inventory of executive function–Preschool version)	1.434, [1.349, 1.520]
Romero López (2018) (Dissertation) Contrast 2	67,19	Typically developing	Executive functions training *EFE – 5* (Group) (*n* = 69)		EF: BRIEF-P	1.206, [1.127, 1.285]
Passolunghi and Costa, 2016 Contrast 1	65,1	Typically developing	WM training (Group) (*n* = 15)	Passive control (*n* = 18)	Early numeracy: ENT (Early numeracy test)	0.390, [0.157, 0.622]
Passolunghi and Costa (2016) Contrast 2	65,235	Typically developing		Active control (*n* = 15)	Early numeracy: ENT	−0.496, [−0.751, −0.240]
Re et al., 2015 study 1	63,225	ADHD symptoms	Executive Functions training *Sviluppare la concentrazione e l'autoregolazione* (Group) (*n* = 13)	Passive control (*n* = 13)	Inattention: PDDAI (Identificazione Precoce del Disturbo da Deficit di Attenzione/iperattività per Insegnanti) (Teacher)	−0.118, [−0.401, 0.165]
					Hyperactivity: IPDDAI (Teacher)	−0.029, [−0.312, 0.254]
Re et al., 2015 study 2	65,38	Typically developing	Executive Functions training *Sviluppare la concentrazione e l'autoregolazione* (Group) (*n* = 13)	Passive control (*n* = 13)	Inattention: PDDAI (Teacher)	−0.626, [−0.923, −0.328]
					Hyperactivity: IPDDAI (Teacher)	−0.036, [−0.319, 0.247]
Rojas-Barahona et al., 2015	52,3	Low SES	WM training (Individual, Computerized) (*n* = 144)	Active control (*n* = 124)	Literacy: ELS (Tejas LEE test)—overall	0.680, [0.649, 0.711]
Salvaguardia et al., 2009	71	ADHD symptoms	WM training *Sviluppare la concentrazione e l'autoregolazione: Giochi e attività sul controllo della memoria di lavoro* (Group) (*n* = 18)	Passive control (*n* = 14)	Inattention: SDAI (Scala di disattenzione e iperattività) (Teacher)	−0.386, [−0.628, −0.145]
					Hyperactivity: SDAI (Teacher)	−0.307, [−0.546, −0.067]
Schmitt et al., 2018 study 1	55,2	Typically developing	Executive functions training using block play (Individual) (*n* = 24)	Passive control (*n* = 35)	Early numeracy: PENS-B (Preschool Early Numeracy Skills Screener -Brief Version)	0.125, [−0.009, 0.259]
Schmitt et al., 2015 study 2	51,645	Low SES	Executive functions training (Group) (*n* = 126)	Passive control (*n* = 150)	Problem behaviors: CBRS (Child behavior rating scale)	−0.047, [−0.076, −0.019]
					Maths: Applied problems	−0.001, [−0.029, 0.027]
					Literacy: Letter/Word identification	0.137, [0.109, 0.166]
Tominey and McClelland, 2011	54,5	Typically developing	Executive functions training *Red Light Purple Light* (Group) (*n* = 28)	Passive control (*n* = 37)	Literacy: Letter/Word identification	0.424, [0.301, 0.547]
					Maths: Applied problems (counting, additions, reading numbers)	0.022, [−0.098, 0.142]
Volckaert and Noël, 2015	60,32	Typically developing	Inhibition training (Group) (*n* = 24)	Active control (*n* = 23)	Problem behaviors: CPRS (Conners parent rating scale)—conduct problems	0.205, [0.043, 0.368]
					Problem behaviors: CPRS—hyperactivity	0.402, [0.237, 0.567]
					Problem behaviors: CPRS—impulsivity	0.351, [0.187, 0.515]
					Problem behaviors: CTRS (Conners teacher rating scale)—conduct problems	−0.164, [−0.326, −0.002]
					Problem behaviors: CTRS—inattention	0.505, [0.339, 0.672]
					Problem behaviors: CTRS—hyperactivity	−0.053, [−0.215, 0.108]

*Italic text indicates the commercial name of that training programs*.

### Inspection for Publication Bias

**Figure 2:**
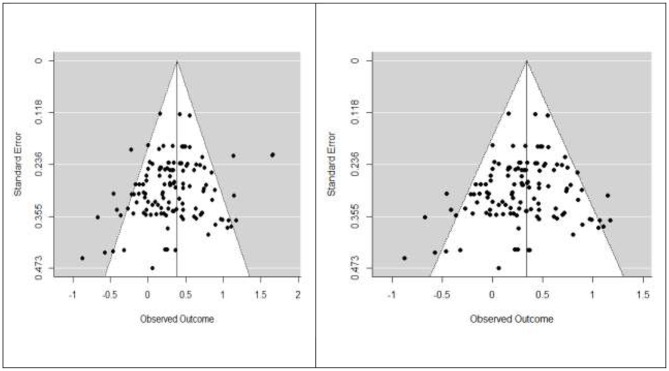
Funnel plot of the meta-analysis of main outcomes of all studies **(left)** and of published studies **(right)**. Each plotted point represents the standard error and standardized mean difference (Hedge's g) between control and Intervention group for a single outcome. The white triangle represents the region where 95% of the data points are expected to lie in the absence of publication bias. The vertical line represents the estimated effect size, based on the meta-analysis.

To investigate for potential publication bias, we explored the funnel plot and checked for differences in effect sizes between published and unpublished studies. The funnel plot is presented in Figure 2, left panel. No evidence of publication bias emerged, Kendall's tau = −0.052, *p* = 0.389. A visual inspection shows that only a few studies fall outside of the triangular region of the pseudo-confidence interval.

Next, we compared the effect sizes of published and unpublished studies, as higher effects for published studies might be an important indication of publication bias. We were able to locate only two unpublished studies, with a total of five different outcomes. No evidence of publication bias emerged. On the contrary, the size of the effect was almost three times bigger for the two unpublished studies than for the published studies: for the unpublished studies the effect was g = 0.949, SE = 0.220, 95% CI = (0.514, 1.383) and for the published studies the effect was g = 0.345, SE = 0.059, 95% CI = (0.227, 0.462). We, therefore, excluded the two unpublished studies from the analyses reported in the manuscript as a conservative strategy to avoid the risk of inflated estimations of the effects. We conducted parallel analyses, including data from the two unpublished studies. These analyses revealed very similar patterns of results and are presented in Supplementary Material. The funnel plot for the dataset of the published studies, on which we conducted the analysis, is presented in Figure 2, right panel. Also in this case, the funnel plot presented no evidence of publication bias, Kendall's tau = −0.0513, *p* = 0.402. A subsequent analysis indicated that the size of the effect was not related to the year of publication of the study (Table 3). Moreover, a sample size moderator analysis was performed, which did not find significant effects (*p* = 0.430), suggesting that differences in sample size are not an important source of the heterogeneity of the results.

**Table 3:** Moderation effects for the primary outcomes of the meta-analysis.

**Effect**	**No. outcomes**	**No. studies**	**Estimated g**	**SE**	**95% CI**		***p*-value**
Year of publication	13	32	0.015	0.017	−0.018	0.049	0.368
**Variables of the children**							
Children's age (months)	120	30	0.007	0.006	−0.006	0.020	0.278
Development at risk	123	32	0.234	0.108	0.019	0.448	0.033
*No-risk*	*108*	*31*	*0.291*	*0.050*	*0.192*	*0.390*	*<0.001*
*Low SES*	*7*	*6*	*0.430*	*0.107*	*0.219*	*0.641*	*<0.001*
*ADHD*	*8*	*8*	*0.785*	*0.169*	*0.451*	*1.120*	*<0.001*
**Variables of the study**							
Control group	123	32	0.054	0.086	−0.116	0.224	0.529
*Passive*	*84*	*23*	*0.360*	*0.055*	*0.251*	*0.468*	*<0.001*
*Active*	*39*	*12*	*0.306*	*0.076*	*0.156*	*0.455*	*<0.001*
Training: computerized	121	32	0.092	0.098	−0.102	0.286	0.102
*Computeriz*.	*59*	*13*	*0.281*	*0.079*	*0.124*	*0.137*	*<0.001*
*Non Comp*.	*62*	*19*	*0.373*	*0.058*	*0.258*	*0.488*	*<0.001*
Training: group	121	32	0.271	0.109	0.055	0.486	0.014
*Individual*	*46*	*17*	*0.211*	*0.056*	*0.055*	*0.343*	*<0.001*
*Group*	*75*	*16*	*0.443*	*0.075*	*0.330*	*0.556*	*<0.001*
Number of sessions	121	32	0.005	0.004	−0.003	0.012	0.225
Length (minutes)	121	32	0.00023	0.00008	0.0006	0.0040	0.008
**Variables of the outcome**							
Near vs. Far training	123	32	0.034	0.068	−0.101	0.169	0.619
*Near*	*76*	*30*	*0.352*	*0.050*	*0.252*	*0.451*	*<0.001*
*Far*	*47*	*16*	*0.318*	*0.068*	*0.186*	*0.449*	*<0.001*

*Italic text indicates the levels of the categorical variables*.

### Main Analyses

#### Overall Effect of EFs Training

A significant overall effect of training of low-to-medium size emerged, g = 0.342, SE = 0.056, *t*_(122)_ = 7.408, *p* < 0.001, 95% CI = (0.252, 0.451). The test for heterogeneity revealed significant variation between effect sizes, Q_(122)_ = 172.340, *p* < 0.001. The log-likelihood tests indicated that the within-study variance and the between-study variance were both significant. The estimated variance between the outcomes within studies was 0.005 and, based on Cheung (2015)'s formulas (see Assink and Wibbelink, 2016), we estimated that it accounted for 12.360% of the variance. The estimated between studies variance was 0.035, and we estimated that it accounted for 47.981% of the variance. The remaining 39.658% of the variance could be attributed to within study sampling variance. In sum, effect sizes varied substantially between studies, but also a modest within study variance emerged. The likelihood ratio indicated that only the between studies variance was significant (LRT = 0.291, *p* = 0.295, and LRT = 7.506, *p* = 0.003, respectively for outcome and for study, both one-sided). Moreover, the 75% rule (Hunter and Schmidt, 1990) suggests that we should inspect heterogeneity if <75% of the total amount of variance can be attributed to within study sampling variance. Therefore, we proceeded to investigate potential moderators, following the research questions outlined above.

#### Investigation of the Potential Moderators

Table 3 reports the results of the tests of the moderators. For categorical moderators, we report the coefficients and tests for the moderation (which indicates the difference between the two categories), and for the intercepts based on each level of the variable (dummy coded, indicating the effect size for each category of the moderator separately). For continuous moderators (meta-regression), the unstandardized regression coefficient and significance for the slope is reported, which indicates the impact of each unitary change in the moderator on the effect size.

Subsequently, we investigated the impact of two moderators related to the children: the mean age of the sample and developmental risk status. The mean age ranged between 51.2 and 74.4 months and did not significantly influence the EFs training effect. We categorized the presence of a developmental risk into three groups: children without developmental risks, children with symptoms of ADHD, and children characterized by low SES. The analysis indicated that the presence of a developmental risk significantly increased the effect of training (*p* = 0.033). The number of studies involving at-risk populations of children, however, was relatively small: we found only four studies, with a total of eight different effects and 112 participants characterized by ADHD, and four studies, with a total of seven effects and 651 participants characterized by low socio-economic status. Subsequent analyses indicated that the effect of EFs training was significant both for children with and without developmental risk. Two comparisons were performed to specifically test for the presence of differences in the effect of EFs training between children with typical development on the one hand, and children from low SES families and children with ADHD symptoms on the other hand. It emerged that the effect of EFs training did not differ significantly between children from low SES and other children without developmental risks (*p* = 0.339). However, a significant difference emerged for the comparison between children with ADHD symptoms and other children in average SES families (*p* = 0.007).

We, next, compared studies with active and passive control groups. The difference in the EFs training effect was non-significant and negligible in terms of effect size. Two characteristics of the training, on the other hand, proved significant: in particular, effects of non-computerized training were twice as big as those of computerized training, and effects of group training were twice as big as those of individual training. However, also in the computerized training and individual training conditions, the effects of training were significant, albeit much smaller in size. On the other hand, the number of sessions effect was not significant, but the overall length of the training significantly influenced its efficacy.

Finally, the comparison between near and far transfer effects showed that both near and far training effects were significant. While the far transfer effect was slightly smaller than the near transfer effect, this difference was not significant.

#### Effect of EF Training on Additional Non-EF Outcomes

Finally, we investigated the transfer of EF training on non-EF outcomes, based on a total of 39 outcomes from 15 studies. The overall effect of training on these effects was not significant and low in size, g = 0.169, SE = 0.106, *t*_(38)_ = 1.583, *p* = 0.122, 95% CI = (−0.047, 0.383).

Three forest plots showing all effect sizes concerning Near Transfer, Far Transfer, and Additional Outcome Measures are presented in Supplementary Material describing.

## Discussion

The aim of the present meta-analysis was to assess the efficacy of cognitive EFs training programs in preschool children aged from 3 to 6 years old. The final dataset consisted of 32 studies published between 2009 and 2019, 21 of which had been published between 2015 and 2019, showing a trend of an increasing number of studies.

We were interested in assessing whether cognitive training could improve EFs in preschool children, comparing near and far transfer effects and analyzing various potential moderators, such as age, presence of developmental risk, and type of cognitive training.

First of all, we found evidence that cognitive training programs are beneficial for EFs in children aged between 3 and 6: the overall effect is medium (*g* = 0.342), but the most interesting results are that near and far transfer effects on EFs were statistically significant and that their sizes were not significantly different.

Near transfer refers to the effect of the cognitive training on the EF measures specifically trained (Working Memory, Inhibitory Control, Cognitive Flexibility, Planning, and Fluid Reasoning, as defined by Diamond, 2013). Far transfer refers to the effect of the training on EFs variables not directly trained. We expected to find a near transfer effect, as many other authors proposed (Diamond and Lee, 2011; Diamond and Ling, 2016) and confirmed in a recent meta-analysis (Kassai et al., 2019). Previous studies did not find far transfer effects on EFs (Melby-Lervåg and Hulme, 2013; Sala and Gobet, 2017; Kassai et al., 2019). The present meta-analysis showed that cognitive EFs training for preschoolers produced both near and far transfer effects, with similar effect sizes (*g* = 0.352 and *g* = 0.318, respectively). Compared to Kassai et al. (2019) we found similar results in terms of near transfer (their g was 0.44), but different considering the far transfer effects (their *g* was 0.11). However, our meta-analysis has important differences compared to the previous ones. First, we included 32 studies specifically focused on preschoolers; secondly, we selected only cognitive training programs, excluding motor-based activities, curriculum based programs, or mindfulness interventions; third, the present meta-analysis was based on a different set of studies (only five papers in our database were present also in the datasets of the previous meta-analyses).

The younger ages of the children of the samples in the present dataset, as compared to the previous meta-analytical studies, is probably the key aspect to explaining the difference of the present results: A far transfer effect is observable in preschoolers probably because their EFs structure is not so well-defined and separate as it becomes at older ages. Accordingly, we assume that cognitive training for improving a specific EF could affect other, not directly trained, EFs because of the intercorrelation and overlap between EFs at this developmental stage. For instance, some authors proposed one single EF factor (Hughes et al., 2010; Wiebe et al., 2011) or two factors (Usai et al., 2014; Scionti and Marzocchi, submitted) and it is, therefore, plausible that a modularization of the EFs is not still completed in preschoolers.

A second important aspect is participants' age: We included the age effect in the analysis to check whether cognitive EFs training was more effective in younger vs. older preschoolers. Age, considered as a continuous variable, was found to be not significant, therefore we conclude that in this age range, a cognitive EF training is similarly effective for younger and older preschool children. Therefore, it is possible that an absence of complete modularization of EF is present also in older preschoolers. The absence of an age effect confirmed the results obtained by other meta-analyses that included older participants (Kassai et al., 2019; Takacs and Kassai, 2019).

In the current research, we found that EFs cognitive training programs in preschoolers are more effective for developmentally-at-risk children than for children without risks. In particular, children with symptoms of ADHD did benefit from EFs training, and the effect size was particularly interesting (*g* = 0.785). Previous studies reported that children with ADHD showed a significant EF improvement after cognitive training, in particular concerning Working Memory (Klingberg et al., 2005; Holmes et al., 2010; Rapport et al., 2013). Cortese et al. (2015) found a small but significant effect of cognitive training in children with ADHD (*d* = 0.37), in particular on inattention symptoms (*d* = 0.47), but not on hyperactivity, concluding that inattention is more malleable to training than hyperactivity. Rapport et al. (2013) focused their meta-analysis on Working Memory training for children with ADHD and found a higher effect (*d* = 0.63) than Cortese et al. (2015). Therefore, we supposed that specific training interventions for Working Memory could be more effective for children with ADHD than a general EF training, since Working Memory difficulties is a key endophenotypes of ADHD (Castellanos and Tannock, 2002). In our study, the effect size of the EF training in children with symptoms of ADHD was even higher (*g* = 0.785) than in Rapport et al. (2013): We suppose that training EFs in preschoolers via a cognitive program could be a useful strategy to improve their neuropsychological skills, in particular in younger children (between 3 and 6 years old) and if they present a disadvantaged condition. Actually, in the current meta-analysis, only 4 studies including ADHD children were present, therefore more research is needed to draw stronger conclusions.

In our study, the effect of cognitive training on EFs development was also significant in children of families with low SES (*g* = 0.430). Other studies demonstrated a similar effect (Blair and Raver, 2014), confirming the hypothesis that an educational program, even in a school setting, for children with socially disadvantaged conditions is important to reduce subsequent psychological risk factors. Although our results confirm the higher effect of the EFs training for children with ADHD or low SES, we found a significant positive effect also in children without developmental risk. This result is encouraging because we hypothesize that cognitive EFs training is useful mostly in an educational context (kindergartner) in order to strengthen the cognitive development and prevent future developmental risk. On this vein, Melby-Lervåg et al. (2016) and Takacs and Kassai (2019) found a significant and positive effect of EFs training on cognitive processes, in particular on working memory in follow-up studies. Future researches could help us to understand whether cognitive EFs training, probably repeated more than once, could help children to reduce possible developmental risks and increase their school and social achievement when they become older.

A controversial issue regarding the EFs cognitive training effect is the comparison between trained and control groups; it is possible that a child would benefit from an EFs training just because s/he receives a cognitive stimulation. If this is the case, it is impossible to disentangle the effect of EFs training from the general and unspecific benefit of being part of a trained group. For this reason, different researches proposed a comparison between a trained and active control group whose participants are involved in other cognitive activities unrelated to EFs. In the current study, a comparison between trained vs. active control group and trained vs. non-active control groups was carried out, assuming that the difference between trained vs. non-active control groups would be higher than the comparison trained vs. active group. Contrary to our expectation, we did not find any difference between the two comparisons. Therefore, we conclude that cognitive EFs training is specifically effective in enhancing cognitive processes and these benefits are not just related to an undifferentiated cognitive stimulation, because the Intervention group demonstrated higher benefit than both the active and passive control groups. A previous meta-analysis on children aged between 2 and 12 years old (Kassai et al., 2019) found similar results, without differentiation between passive and active control groups.

According to the literature, a promising way to improve EFs in children is related to the use of computerized programs, probably because computerized training, for children, could be as motivating as playing a videogame. As Martinovic et al. (2016) demonstrated, videogames are engaging if they are simple and rewarding, but they are not motivating if they ask the children to improve their attention and problem-solving skills. Moreover, in their meta-analysis concerning computerized EF training programs, Webb et al. (2018) found a small effect on the three EF factors (Inhibition, Updating, and Shifting): Hedges' *g* effect size ranged from 0.005 (Updating) to 0.16–0.17 (Shifting and Inhibition). It is important to note, however, that Webb et al. (2018) analyzed a large sample of participants, mostly older adults, probably not very familiar to work with a computer: For this reason, they are, most probably, not the best target for a computerized training. In our study, we did not find a significant difference between computerized and non-computerized training. Although the average effect of the computerized training was higher than in the work of Webb et al. (2018) (current study = 0.281; Webb = 0.17), we found a non-significant (*p* = 0.10) higher benefit for non-computerized training (*g* = 0.373). Therefore, as underlined by Diamond and Ling (2016), computerized training probably could be effective only for the Inhibition component of EFs. In other words, playing with cards, doing body exercises, and paper and pencil activities could be more effective for improving EFs than using a tablet or a computer, but the available empirical evidence does not allow to draw a firm conclusion on this point.

A further comparison was made to investigate whether EF training could be more effective if presented in a group or individually. Both conditions have pros and cons: For preschoolers a group could be more motivating and fun, but less specific and, in some cases, more confusing. According to our results, cognitive training is more effective if administered in groups than individually, contrary to what Moreau and Conway (2014) proposed about individualized training tailored to one's particular needs and expectations. Both conditions can produce positive effects on EFs development, but in the group, the profit of cognitive training is more than double than in individually administered training. Actually, Moreau and Conway (2014) underlined the importance of individualized training for the general population, while our study was focused on preschoolers, that probably gain more in group and in individualized training. Many training programs for preschoolers are presented in a group format in kindergartners, and the positive effect of this condition has been already demonstrated (e.g., Röthlisberger et al., 2012; Kassai et al., 2019). In terms of cost/benefits ratio, it is encouraging to know that group-administered cognitive training is even more effective, because it could be proposed both at school and in clinical settings, saving up economic resources without reducing its efficacy.

According to previous studies (Ericsson, 2009), training length was supposed to be related to higher improvement of EFs. Our results partly support this hypothesis; if we consider the number of sessions the positive effect is not significant, but if we consider the total amount of training time there is a positive and significant association. This result is consistent with other results reported in a previous meta-analysis (Takacs and Kassai, 2019) and it underlines the importance of the minimum amount of time before observing significant improvement in the EFs development, and that benefits need exercise.

A final comment concerns the effect of the cognitive training on the additional outcomes (see forest plot in Supplementary Material): the effect size was not significant (*g* = 0.10) confirming the difficulty to generalize from a cognitive EF training to other psychological domains, such as learning prerequisites and behavioral aspects.

In summary, the current meta-analysis on cognitive training for enhancing EFs in preschool children showed positive and significant results in terms of benefits for psychological development. This is the first meta-analysis on EF cognitive training for preschoolers: As hypothesized, we found a positive and significant effect concerning near and far transfer effects on Executive Functioning. Positive effects of EF training programs were significant for children with or without developmental risks. Moreover, cognitive EFs training programs are more effective if administered in group.

## Limitations

The current meta-analysis has some limitations: firstly, the far transfer effect could also be due to task impurity, because tests for preschoolers usually activate multiple cognitive processes, and we cannot exclude that some tasks aimed to assess far EFs effect, actually assess partly near EFs. Secondly, there was considerable variability in the size of the samples included in the studies. Despite the exclusion of studies with less than eight participants per group, some of the studies included in the analysis still have small sample sizes, which might potentially overestimate treatment effects. We found no evidence of a relationship between sample size and EFs effect, but we nevertheless urge the reader to be cautious before drawing strong conclusions about the effects of the cognitive EFs training in preschoolers. Thirdly, we tried to include unpublished studies, but their atypical results (extremely large size of the effects) could bias the results, therefore, we decided to exclude these two analysis. Finally, we found few studies on preschool children with developmental risks and only one type of neurodevelopmental disorder, namely ADHD. We did not find studies on EFs training for preschoolers with other neurodevelopmental disorders (autism, language impairment, motor coordination disorder), although an EFs impairment has been demonstrated in these groups. Future research is necessary to assess whether cognitive EFs training could be useful with children presenting other neurodevelopmental disorders.

## Data Availability Statement

All datasets generated for this study are included in the article/Supplementary Material.

## Author Contributions

NS and MC contributed to conceptualization, design and methodology of the study and data collection, discussed interpretation of results, and wrote part of the manuscript. CZ contributed to conceptualization, design and methodology of the study, was responsible for outcome assessments and data collection, carried out data analyses and interpretation of data, and wrote part of the manuscript. GM contributed to conceptualization of the study, supervised data collection, was responsible for interpretation of data, and wrote part of the manuscript. All authors gave final approval of the version to be published and agreed to be accountable for all aspects of the work.

### Conflict of Interest

The authors declare that the research was conducted in the absence of any commercial or financial relationships that could be construed as a potential conflict of interest.

## Appendix A

“preschoolers” OR “preschool” OR “early childhood” OR “3-6 years”

AND

“training” OR “therapy” OR “intervention^*^” OR “program” OR “treatment”

AND

“executive function^*^” OR “attention^*^” OR “working memory^*^” OR “updating” OR “inhibitory control” OR “self-regulation” OR “self-regulation” OR “cognitive flexibility” OR “mental flexibility” OR “shifting” OR “set shifting” OR “effortful control” OR “cognitive control” OR “problem solving” OR “planning” OR “executive control” OR “metacognition” OR “behavioral control” OR “self-control” OR “response inhibition” OR “interference control” OR “executive attention” OR “focused attention” OR “selective attention”
