# Corrigendum: Is Cognitive Training Effective for Improving Executive Functions in Preschoolers? A Systematic Review and Meta-Analysis

**DOI:** 10.3389/fpsyg.2020.00410

**Published:** 2020-03-24

**Authors:** Nicoletta Scionti, Marina Cavallero, Cristina Zogmaister, Gian Marco Marzocchi

**Affiliations:** Department of Psychology, University of Milan – Bicocca, Milan, Italy

**Keywords:** executive functions, EF training, cognitive training, preschoolers, meta-analysis

In the original article, there was an error because we wrongly coded four studies (Gade et al., 2017) as including a computerized training, which was not correct. In the Abstract, we stated that the condition computerized vs. non-computerized versions of the training was significant, but this was not the case. Moreover, in Table 1, we deleted “computerized” in the four studies published by Gade et al. (2017) and on Table 3, we corrected the values related to “computerized vs. non-computerized trainings”. Supplementary Data Sheet 2 has been corrected and updated. Corrections have also been made to Table 1, Table 3, as well as the following paragraphs indicated below:

**Table 1:** Summary of the studies included into the meta-analysis: EF outcome measures and Near vs. Far Transfer effects.

**References**	**Mean age (in months)**	**Clinical risk status of sample**	**Training condition**	**Number of session**	**Control condition**	**Executive outcome measure**	**Type of transfer**	**Hedge's g, [95% CI]**
Gade et al., 2017	62,39	Typically developing	Visuo-spatial WM training (Individual)	11	Active control (*n* = 10)	Verbal WM		
Study 1			(*n* = 10)			- Word span	Far transfer	0.230, [−0.132, 0.592]
						Visuospatial WM		
						- Matrix span	Near transfer	0.225, [−0.137, 0.587]
						- Object span task	Near transfer	0.361, [−0.005, 0.727]
Gade et al., 2017	67,19	Typically developing	Visuo-spatial WM training (Individual)	12,5	Active control (*n* = 16)	Verbal WM		
Study 2			(*n* = 15)			- Word span	Far transfer	0.643, [0.390, 0.896]
						Visuospatial WM		
						- Matrix span	Near transfer	0.108, [−0.133, 0.348]
						- Color span backward	Near transfer	−0.364, [−0.609, −0.120]
Gade et al., 2017	72	Typically developing	Visuo-spatial WM training (Individual)	13,5	Active control (*n* = 10)	Verbal WM		
Study 3			(*n* = 10)			- Word span	Far transfer	0.257, [−0.106, 0.619]
						Visuospatial WM		
						- Matrix span	Near transfer	−0.319, [−0.683, 0.046]
						- Color span backward	Near transfer	0.368, [0.002, 0.735]
Gade et al., 2017	61,3	Typically developing	Visuo-spatial WM training (Individual)	12	Active control (*n* = 10)	Verbal WM		
Study 4			(*n* = 10)			- Word span	Far transfer	−0.463, [−0.833, −0.093]
						Visuospatial WM		
						- Matrix span	Near transfer	−0.875, [−1.273, −0.478]

**Table 3:** Moderation effects for the primary outcomes of the meta-analysis.

**Effect**	**No. outcomes**	**No. studies**	**Estimated g**	**SE**	**95% CI**		***p*-value**
Training: computerized	121	32	0.092	0.098	−0.102	0.286	0.102
*Computeriz*.	*59*	*13*	*0.281*	*0.079*	*0.124*	*0.137*	*<0.001*
*Non Comp*.	*62*	*19*	*0.373*	*0.058*	*0.258*	*0.488*	*<0.001*

**The Abstract**

“In the present meta-analysis, we examined the effect of cognitive training on the Executive Functions (EFs) of preschool children (age range: 3–6 years). We selected a final set of 32 studies from 27 papers with a total sample of 123 effect sizes. We found an overall effect of cognitive training for improving EF (*g* = 0.352; k = 123; *p* < 0.001), without significant difference between near and far transfer effects on executive domains. No significant additional outcome effects were found for behavioral- and learning-related outcomes. Cognitive training programs for preschoolers are significantly more effective for developmentally at-risk children (ADHD or low socio-economic status) than for children with typical development and without risks. Other significant moderators were: individual vs. group sessions and length of training. The number of sessions and computerized vs. non-computerized training were not significant moderators. This is the first demonstration of cognitive training for transfer effects among different executive processes. We discuss this result in relationship to the lower level of modularization of EFs in younger children.”

In the Discussion, paragraph 10 and final paragraph, we reported two modifications according to the corrections. The new data analysis revealed that the difference between computerized vs. non-computerized training was no longer significant and only a trend of significance (*p* = 0.10) was present.

**The Discussion, Paragraph 10**

“According to the literature, a promising way to improve EFs in children is related to the use of computerized programs, probably because computerized training, for children, could be as motivating as playing a videogame. As Martinovic et al. (2016) demonstrated, videogames are engaging if they are simple and rewarding, but they are not motivating if they ask the children to improve their attention and problem-solving skills. Moreover, in their meta-analysis concerning computerized EF training programs, Webb et al. (2018) found a small effect on the three EF factors (Inhibition, Updating, and Shifting): Hedges' *g* effect size ranged from 0.005 (Updating) to 0.16–0.17 (Shifting and Inhibition). It is important to note, however, that Webb et al. (2018) analyzed a large sample of participants, mostly older adults, probably not very familiar to work with a computer: For this reason, they are, most probably, not the best target for a computerized training. In our study, we did not find a significant difference between computerized and non-computerized training. Although the average effect of the computerized training was higher than in the work of Webb et al. (2018) (current study = 0.281; Webb = 0.17), we found a non-significant (*p* = 0.10) higher benefit for non-computerized training (*g* = 0.373). Therefore, as underlined by Diamond and Ling (2016), computerized training probably could be effective only for the Inhibition component of EFs. In other words, playing with cards, doing body exercises, and paper and pencil activities could be more effective for improving EFs than using a tablet or a computer, but the available empirical evidence does not allow to draw a firm conclusion on this point.”

**The Discussion, Final paragraph**

“In summary, the current meta-analysis on cognitive training for enhancing EFs in preschool children showed positive and significant results in terms of benefits for psychological development. This is the first meta-analysis on EF cognitive training for preschoolers: As hypothesized, we found a positive and significant effect concerning near and far transfer effects on Executive Functioning. Positive effects of EF training programs were significant for children with or without developmental risks. Moreover, cognitive EFs training programs are more effective if administered in group.”

The authors apologize for this error and state that this does not change the scientific conclusions of the article in any way. The original article has been updated.
