# Supplementary material for: Is Cognitive Training Effective for Improving Executive Functions in Preschoolers? A Systematic Review and Meta-Analysis
Source: Front Psychol. 2020 Jan 10;10:2812. doi: 10.3389/fpsyg.2019.02812 (PMC6965160; doi:10.3389/fpsyg.2019.02812)
Supplement: Supplementary file 1 [file Table_1.PDF]

Results of the Parallel Analysis conducted on all studies that met our criteria, including the two unpublished papers.

A significant overall effect of training of low-to-medium size emerged, estimated g = 0.388, SE = 0.063, t(127) = 6.152, p < .001, 95% CI = (0.263 0.513) . The test for heterogeneity revealed significant variation between effect sizes, Q(127) = 269.917, p < .001. The log-likelihood tests indicated that the within-study variance and the between-study variance were both significant. The variance between the effect sizes within studies was estimated as 0.023 and it accounted for 12.499% of the variance. The variance between studies was 0.091 and it accounted for 48.516% of the variance. The likelihood ratio indicated that both sources of variance were significant (LRT = 4.782, p = .014, and LRT = 18.499, p < .001 respectively, both one-sided). The remaining 38.985% of the variance could be attributed to within study sampling variance. In sum, effect sizes varied substantially between studies, but also a modest and significant within study variance emerged.

The following Tables report the estimated effects of the moderators. All studies (published and unpublished).

Table 1. Characteristics of the publication

Effect	No. outcomes	Effect size g	SE	95% CI		p-value
				Lower limit	Upper limit	
Publication status	128	0.604	0.227	0.154	1.054	.009
Published	*123*	*0.345*	*0.059*	*0.227*	*0.462*	*< .001*
Unpublished	*5*	*0.949*	*0.220*	*0.514*	*1.383*	*< .001*
Year of pub.	128	0.030	0.023	-0.017	0.076	.206

Table 2. Characteristics of the participants.

Effect	No. outcomes	Effect size g	SE	95% CI		p-value
Age (months)	123	0.006	0.006	-0.005	0.018	.318
Development	128	0.213	0.156	-0.095	0.524	.173
Typical	*113*	*0.386*	*0.071*	*0.206*	*0.486*	*< .001*
Low SES	*7*	*0.455*	*0.169*	*0.121*	*0.788*	*< .001*
ADHD	*8*	*0.793*	*0.224*	*0.351*	*1.236*	*< .001*

Table 3. Characteristics of the control groups.

Effect	No. outcomes	Effect size g	SE	95% CI		p-value
Control group	128	0.004	0.103	-0.200	0.208	.967
Passive	*87*	*0.390*	*0.073*	*0.246*	*0.533*	*< .001*
Active	*41*	*0.386*	*0.093*	*0.201*	*0.570*	*< .001*

Table 4. Characteristics of the training and of the outcome

Effect	No. outcomes	Effect size g	SE	95% CI		p-value
Training: computerized	126	0.304	0.121	0.065	0.543	.013
Computerized	68	0.208	0.092	0.025	0.390	.026
Non computerized	58	0.512	0.078	0.358	0.666	< .001
Training: group	126	0.271	0.109	0.055	0.486	.014
Individual	*51*	*0.244*	*0.081*	*0.085*	*0.404*	*.003*
Group	*75*	*0.515*	*0.078*	*0.361*	*0.669*	*< .001*
Number of sessions	126	0.001	0.003	-0.005	0.007	.688
Length (minutes)	126	0.0001	0.00009	-0.0006	0.0003	.207
**Variables of the outcome**						
Near vs. Far	128	0.108	0.075	-0.041	0.256	.153
Near	*79*	*0.421*	*0.066*	*0.289*	*0.552*	*< .001*
Far	*49*	*0.313*	*0.075*	*0.152*	*0.474*	*< .001*

No additional outcomes were present in the unpublished studies.

Parallel analyses - excluding papers in languages other than English (i.e., Italian and Spanish)

Note that the papers in other languages are four, two of which were published and two unpublished. The analysis reported hereafter shows that no major difference emerged between the results of analyses conducted on all data (reported in the main paper) and analyses conducted on data from manuscripts in English.

Considering only papers in English language, thirty studies were eligible for inclusion, for a total of 121 different outcomes, with 955 participants in the training, 333 participants in the active control, and 703 in the passive control conditions.

Inspection for publication bias

To investigate for potential publication bias, we explored the funnel plot (note that the difference between published and unpublished studies could not be tested, as only published studies were found in the English language dataset). The funnel plot is presented in Figure 1. No evidence of publication bias emerged, Kendall's tau = -.052, p = .389. A visual inspection shows that only a few studies fall outside of the triangular region of the pseudo-confidence interval.

Figure 1. Funnel plot for the studies in English language.

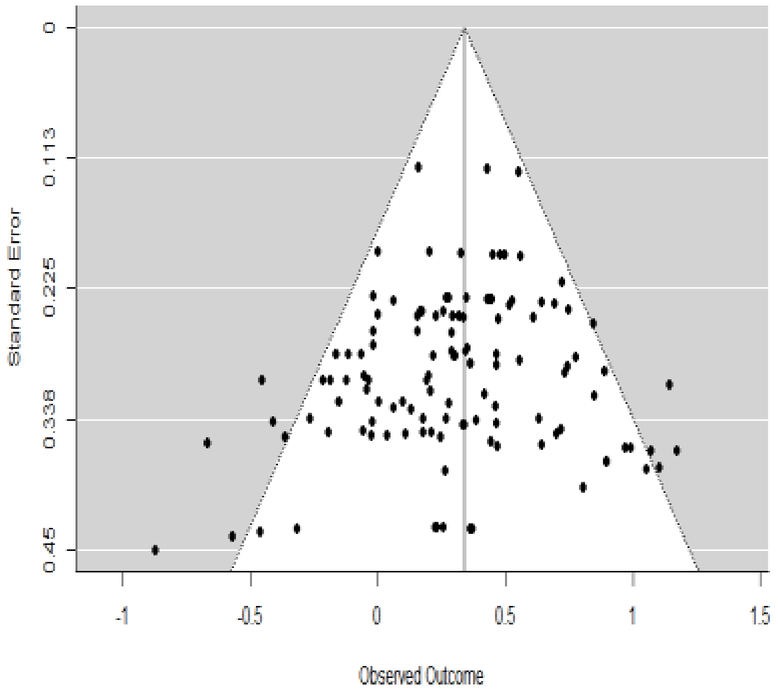

Main Analyses

Overall effect of EF training. A significant overall effect of training of low-to-medium size emerged, g = 0.340, SE = 0.047, t(120) = 7.233, p < .001, 95% CI = (0.247,0.434). The test for heterogeneity revealed significant variation between effect sizes, Q(120) = 171.838, p = .001. The estimated variance between the outcomes within studies was 0.006 and, based on Cheung (2014)'s formulas (see Assink & Wibbelink, 2016), we estimated that it accounted for 5.042 % of the variance. The estimated between studies variance was 0.036, and we estimated that it accounted for 30.918% of the variance. The remaining 64.040% of the variance could be attributed to within study sampling variance. In sum, effect sizes varied substantially between studies, and a modest within study variance emerged. The likelihood ratio indicated that only the between studies variance was significant (LRT = 0.369, p = .272, and LRT = 7.370, p = .003 respectively for outcome and for study, both one-sided). Moreover, the 75% rule (Hunter & Schmidt, 1990) suggests that we should

inspect heterogeneity if less than 75% of the total amount of variance can be attributed to within study sampling variance. Therefore, we proceeded to investigate potential moderators, following the research questions outlined above.

Table 5. Moderation effects for the primary outcomes of the meta-analysis. Results of the analyses restricted to the English literature.

**Effect**	**No. outcomes**	**No. studies**	**Estimated g**	**SE**	**95% CI**		**p-value**
Year of publication	121	30	0.019	0.018	-0.016	0.054	.283
			**Variables of the children**				
Children's age (months)	118	28	0.006	0.007	-0.006	0.019	.322
Development at risk	121	30	0.228	0.112	0.005	0.450	.045
No-risk	*107*	*22*	*0.293*	*0.049*	*0.196*	*0.389*	*< .001*
Low SES	*7*	*4*	*0.432*	*0.108*	*0.217*	*0.647*	*< .001*
ADHD	*7*	*4*	*0.824*	*0.188*	*0.452*	*1.197*	*< .001*

**Variables of the study**							
Control group	121	30	0.054	0.087	-0.118	0.226	.534
Passive	*82*	*21*	*0.359*	*0.056*	*0.248*	*0.479*	*< .001*
Active	*39*	*10*	*0.305*	*0.076*	*0.154*	*0.455*	*< .001*
Training: computerized	119	30	0.210	0.089	0.033	0.387	.021
Computeriz.	*67*	*13*	*0.221*	*0.066*	*0.089*	*0.352*	*.001*
Non Comp.	*52*	*17*	*0.431*	*0.060*	*0.312*	*0.549*	*< .001*
Training: group	119	31	0.205	0.080	0.046	0.364	.012
Individual	*45*	*16*	*0.235*	*0.056*	*0.123*	*0.346*	*< .001*
Group	*74*	*15*	*0.440*	*0.058*	*0.324*	*0.555*	*< .001*
Number of sessions	119	30	0.005	0.004	-0.003	0.013	.243
Length (minutes)	119	30	0.00022	0.00009	0.0005	0.0039	.012

			**Variables of the outcome**				
Near vs. Far training	121	30	0.033	0.069	-0.104	0.169	.636
Near	*74*	*28*	*0.350*	*0.051*	*0.248*	*0.452*	*< .001*
Far	*47*	*16*	*0.317*	*0.067*	*0.185*	*0.450*	*< .001*
Far 1 vs. Far2 training	47	16	0.141	0.136	-0.132	0.415	.303
Near_2 = 0	*36*		*0.288*	*0.089*	*0.108*	*0.468*	*0.002*
Near_2 = 1	*11*		*0.430*	*0.126*	*0.177*	*0.683*	*0.001*

Investigation of the potential moderators. Table 5 reports the results of the tests of the moderators. For categorical moderators, we report the coefficients and tests for the moderation (which indicates the difference between the two categories), and for the intercepts based on each level of the variable (dummy coded, indicating the effect size for each category of the moderator separately). For continuous moderators (meta-regression), the unstandardized regression coefficient and significance for the slope is reported, which indicates the impact of each unitary change in the moderator on the effect size.

We investigated the impact of two moderators related to the children: the mean age of the sample and developmental risk status. The mean age ranged between 51.2 and 74.4 months and did not significantly influence the EF training effect. We categorized the presence of a developmental risk into three groups: children without developmental risks, children with symptoms of ADHD, and children characterized by low SES. The analysis indicated that the presence of a developmental risk significantly increased the effect of training ($p = .045$). Subsequent analyses indicated that the effect of EF training was significant both for children with and without developmental risk, but it was significantly stronger for children with ADHD symptoms, $p < .02$. The increase in the effect of EF training for children from low SES families was not significant, $p = .35$.

We, next, compared studies with active and passive control groups. The difference in the EF training effect was non-significant and negligible in terms of effect size. Two characteristics of the training, on the other hand, proved significant: in particular, effects of non-computerized training were twice as big as those of computerized training, and effects of group training were twice as big as those of individual training. However, also in the computerized training and individual training conditions, the effects of training were significant, albeit much smaller in size. On the other hand, the number of sessions effect was not significant, but the overall length of the training significantly influenced its efficacy.

Finally, the comparison between near and far transfer effects showed that both near and far training effects were significant. Albeit the far transfer effect was slightly smaller than the near transfer effect, this difference was not significant.

We further differentiated far transfer effects among far transfer among the visuo-spatial and verbal components of working memory (namely Near_2= 1) and the far transfer from one EF to other EFs (e.g. Inhibitory Control transfer to Fluid Reasoning, Planning and WM) (namely Near_2= 0). The difference in the effect for the transfer from visuo-spatial to verbal WM and the transfer from a EF to another effects from was not significant, but due the small number of effects categorized as far transfer among verbal and visuo-spatial WM (Near_2 = 1) we refrain from drawing any conclusion from this absence of significant differences. What is more important for the present purposes, as Table 5 (final two lines) shows, the effects of training were significant both on far transfer among the two component of WM and far transfer among different EFs (e.g. the transfer from Working Memory to Fluid Reasoning, Planning or Inhibitory Control)

Effect of EF training on additional non-EF outcomes. Finally, we investigated the transfer of EF training on non-EF outcomes, based on a total of 35 outcomes from 13 studies. The overall effect of training on these effects was not significant and low in size, g = 0.115, SE = 0.063, t(38) = 1.824, p = .077, 95% CI = (-0.013, 0.242).
