# Supplementary material for: Is Cognitive Training Effective for Improving Executive Functions in Preschoolers? A Systematic Review and Meta-Analysis
Source: Front Psychol. 2020 Jan 10;10:2812. doi: 10.3389/fpsyg.2019.02812 (PMC6965160; doi:10.3389/fpsyg.2019.02812)
Supplement: Supplementary file 2 [file Table_2.PDF]

```
# based on
# Knapp, G., & Hartung, J. (2003). Improved tests for a random effects meta-regression with a single covariate. Statistics in medicine, 22(17), 2693-2710.
# We used the syntax for the purposes of the present meta-analysis and we make it available for transparency of our analyses


library(metafor) #I load the package for the analyses

dataset<-DatiMeta

# I compute the variance of the effectsize
dataset$vd <- (dataset$GSn+dataset$GCn)/(dataset$GSn*dataset$GCn)+ dataset$cohens_d^2/(2*(dataset$GSn+dataset$GCn))
dataset$v<-dataset$CorrectFactJ^2*dataset$vd

# I dichotomize the variable AddOutco
dataset$AddOutco[(dataset$AddOutco=="Reading")]<-"1"
dataset$AddOutco[(dataset$AddOutco=="Math")]<-"1"
dataset$AddOutco[is.na(dataset$AddOutco)]<-"0"
dataset$AddOutco<-as.numeric(dataset$AddOutco)

table(dataset$AddOutco)

#number of participants per comparison

dataset$totalsample<-dataset$GCn + dataset$GSn
summary (dataset$totalsample)

#Here I create the subsample of all FE outcomes (i.e., not "additional" outcomes)
row_to_keep <- which(dataset$AddOutco==0)
dataset<-dataset[row_to_keep,]

#Here I create the subsample of all FE outcomes in published studies
row_to_keep <- which(dataset$Grigia==0)
dataset2<-dataset[row_to_keep,]


#Hereafter I perform the analyses with all studies (last letter = A) and wit only published studies (last letter = B).

overallA <- rma.mv(y, v, random = list(~ 1 | effectsizeID, ~ 1 | studyID), tdist=TRUE, data=dataset)
summary(overallA, digits=3)

# Build a two-level model without within-study variance.
modelnovar2A <- rma.mv(y, v, random = list(~ 1 | effectsizeID, ~ 1 | studyID), sigma2=c(0,NA), tdist=TRUE, data=dataset)

#Perform a likelihood-ratio-test to determine the
# significance of the within-study variance.
anova(overallA,modelnovar2A) # note that the output is two-sided but this test is better performed one-sided, hence the p value must be divided by two.


# Build a two-level model without between-study variance;
# Perform a likelihood-ratio-test to determine the
# significance of the between-study variance.
modelnovar3A <- rma.mv(y, v, random = list(~ 1 | effectsizeID, ~ 1 | studyID), sigma2=c(NA,0), tdist=TRUE, data=dataset)
anova(overallA,modelnovar3A) # note that the output is two-sided but this test is better performed one-sided, hence the p value must be divided by two.

# Determining how the total variance is distributed over the
# three levels of the meta-analytic model;
# Print the results in percentages on screen.
n <- length(dataset$v)
list.inverse.variances <- 1 / (dataset$v)
sum.inverse.variances <- sum(list.inverse.variances)
squared.sum.inverse.variances <- (sum.inverse.variances) ^ 2
list.inverse.variances.square <- 1 / (dataset$v^2)
sum.inverse.variances.square <-
  sum(list.inverse.variances.square)
numerator <- (n - 1) * sum.inverse.variances
denominator <- squared.sum.inverse.variances -
  sum.inverse.variances.square
estimated.sampling.variance <- numerator / denominator
I2_1 <- (estimated.sampling.variance) / (overallA$sigma2[1]
                                         + overallA$sigma2[2] + estimated.sampling.variance)
I2_2 <- (overallA$sigma2[1]) / (overallA$sigma2[1]
                               + overallA$sigma2[2] + estimated.sampling.variance)
I2_3 <- (overallA$sigma2[2]) / (overallA$sigma2[1]
                               + overallA$sigma2[2] + estimated.sampling.variance)
amountvariancelevel1 <- I2_1 * 100
amountvariancelevel2 <- I2_2 * 100
amountvariancelevel3 <- I2_3 * 100
amountvariancelevel1
amountvariancelevel2
amountvariancelevel3




funnel(overallA)


############



overallB <- rma.mv(y, v, random = list(~ 1 | effectsizeID, ~ 1 | studyID), tdist=TRUE, data=dataset2)
summary(overallB, digits=3)

# Build a two-level model without within-study variance.
modelnovar2B <- rma.mv(y, v, random = list(~ 1 | effectsizeID, ~ 1 | studyID), sigma2=c(0,NA), tdist=TRUE, data=dataset2)

#Perform a likelihood-ratio-test to determine the
# significance of the within-study variance.
anova(overallB,modelnovar2B) # note that the output is two-sided but this test is better performed one-sided, hence the p value must be divided by two.
```

```
# Build a two-level model without between-study variance;
# Perform a likelihood-ratio-test to determine the
# significance of the between-study variance.
modelnovar3B <- rma.mv(y, v, random = list(~ 1 | effectsizeID, ~ 1 | studyID), sigma2=c(NA,0), tdist=TRUE, data=dataset2)
anova(overallB,modelnovar3B) # note that the output is two-sided but this test is better performed one-sided, hence the p value must be divided by two.

# Determining how the total variance is distributed over the
# three levels of the meta-analytic model;
# Print the results in percentages on screen.
n <- length(dataset2$v)
list.inverse.variances <- 1 / (dataset2$v)
sum.inverse.variances <- sum(list.inverse.variances)
squared.sum.inverse.variances <- (sum.inverse.variances) ^ 2
list.inverse.variances.square <- 1 / (dataset2$v^2)
sum.inverse.variances.square <-
  sum(list.inverse.variances.square)
numerator <- (n - 1) * sum.inverse.variances
denominator <- squared.sum.inverse.variances -
  sum.inverse.variances.square
estimated.sampling.variance <- numerator / denominator
I2_1 <- (estimated.sampling.variance) / (overallB$sigma2[1]
                                         + overallB$sigma2[2] + estimated.sampling.variance)
I2_2 <- (overallB$sigma2[1]) / (overallB$sigma2[1]
                                + overallB$sigma2[2] + estimated.sampling.variance)
I2_3 <- (overallB$sigma2[2]) / (overallB$sigma2[1]
                                + overallB$sigma2[2] + estimated.sampling.variance)
amountvariancelevel1 <- I2_1 * 100
amountvariancelevel2 <- I2_2 * 100
amountvariancelevel3 <- I2_3 * 100
amountvariancelevel1
amountvariancelevel2
amountvariancelevel3



funnel(overallB)




#Analysis on Grey vs. published



table(dataset$Grigia)

GRIGIA <- rma.mv(y, v, mods = ~ Grigia, random = list(~ 1 | effectsizeID, ~1 | studyID), tdist=TRUE, data=dataset)
summary(GRIGIA, digits=3)

dataset$published<-1-dataset$Grigia

PUBLISHED <- rma.mv(y, v, mods = ~ published, random = list(~ 1 | effectsizeID, ~1 | studyID), tdist=TRUE, data=dataset)
summary(PUBLISHED, digits=3)


######################################


#Any effect of year of publication?

YEARPUB_A <- rma.mv(y, v, mods = ~ year, random = list(~ 1 | effectsizeID, ~
                                                        1 | studyID), tdist=TRUE, data=dataset)
summary(YEARPUB_A, digits=3)

YEARPUB_B <- rma.mv(y, v, mods = ~ year, random = list(~ 1 | effectsizeID, ~
                                                       1 | studyID), tdist=TRUE, data=dataset2)

summary(YEARPUB_B, digits=3)


###########characteristics of the children



#effects of age of the children

dataset$etabimbi <- (dataset$GS_Meta+dataset$GCMeta)/2
summary(dataset2$etabimbi)

ETABIMBIA <- rma.mv(y, v, mods = ~ etabimbi, random = list(~ 1 | effectsizeID, ~ 1 | studyID), tdist=TRUE, data=dataset)
summary(ETABIMBIA, digits=5)


dataset2$etabimbi <- (dataset2$GS_Meta+dataset2$GCMeta)/2
summary(dataset2$etabimbi)

ETABIMBIB <- rma.mv(y, v, mods = ~ etabimbi, random = list(~ 1 | effectsizeID, ~ 1 | studyID), tdist=TRUE, data=dataset2)
summary(ETABIMBIB, digits=5)



#Any effect of typical/atypical development?
table(dataset2$SvAt)

SVATA <- rma.mv(y, v, mods = ~ SvAt, random = list(~ 1 | effectsizeID, ~
1 | studyID), tdist=TRUE, data=dataset)
summary(SVATA, digits=3)



SVATB <- rma.mv(y, v, mods = ~ SvAt, random = list(~ 1 | effectsizeID, ~
                                                     1 | studyID), tdist=TRUE, data=dataset2)
summary(SVATB, digits=3)
```

```
####################

table(dataset$GrAt)
dataset$ADHD<-NA
dataset$DemRis<-NA
dataset$SvTyp<-NA
dataset$ADHD<-0
dataset$DemRis<-0
dataset$SvTyp<-1-dataset$SvAt
dataset$ADHD[dataset$GrAt=="ADHD"]<-1
dataset$DemRis[dataset$GrAt=="DemRis"]<-1
table(dataset$ADHD, dataset$DemRis)

dataset2$ADHD<-NA
dataset2$DemRis<-NA
dataset$SvTyp<-NA
dataset2$ADHD<-0
dataset2$DemRis<-0
dataset2$SvTyp<-1-dataset2$SvAt
dataset2$ADHD[dataset2$GrAt=="ADHD"]<-1
dataset2$DemRis[dataset2$GrAt=="DemRis"]<-1
table(dataset2$ADHD, dataset2$DemRis)




SVAT.A <- rma.mv(y, v, mods = ~ ADHD+DemRis, random = list(~ 1 | effectsizeID, ~
                                                              1 | studyID), tdist=TRUE, data=dataset)
summary(SVAT.A, digits=3)


SVAT.A1 <- rma.mv(y, v, mods = ~ ADHD+SvTyp, random = list(~ 1 | effectsizeID, ~
                                                              1 | studyID), tdist=TRUE, data=dataset)
summary(SVAT.A1, digits=4)


SVAT.A2 <- rma.mv(y, v, mods = ~ DemRis+SvTyp, random = list(~ 1 | effectsizeID, ~
                                                                1 | studyID), tdist=TRUE, data=dataset)
summary(SVAT.A2, digits=4)





SVAT.B <- rma.mv(y, v, mods = ~ ADHD+DemRis, random = list(~ 1 | effectsizeID, ~
                                                             1 | studyID), tdist=TRUE, data=dataset2)
summary(SVAT.B, digits=3)


SVAT.B1 <- rma.mv(y, v, mods = ~ ADHD+SvTyp, random = list(~ 1 | effectsizeID, ~
                                                             1 | studyID), tdist=TRUE, data=dataset2)
summary(SVAT.B1, digits=4)


SVAT.B2 <- rma.mv(y, v, mods = ~ DemRis+SvTyp, random = list(~ 1 | effectsizeID, ~
                                                               1 | studyID), tdist=TRUE, data=dataset2)
summary(SVAT.B2, digits=4)


#Effects of active vs. passive control group

GCATTIVOA <- rma.mv(y, v, mods = ~ GCattivo, random = list(~ 1 | effectsizeID, ~
1 | studyID), tdist=TRUE, data=dataset)
summary(GCATTIVOA, digits=3)

dataset$GCpassivo<-1-dataset$GCattivo
GCPASSIVOA <- rma.mv(y, v, mods = ~ GCpassivo, random = list(~ 1 | effectsizeID, ~
                                                            1 | studyID), tdist=TRUE, data=dataset)
summary(GCPASSIVOA, digits=3)



GCATTIVOB <- rma.mv(y, v, mods = ~ GCattivo, random = list(~ 1 | effectsizeID, ~
                                                            1 | studyID), tdist=TRUE, data=dataset2)
summary(GCATTIVOB, digits=3)

dataset2$GCpassivo<-1-dataset2$GCattivo
GCPASSIVOB <- rma.mv(y, v, mods = ~ GCpassivo, random = list(~ 1 | effectsizeID, ~
                                                              1 | studyID), tdist=TRUE, data=dataset2)
summary(GCPASSIVOB, digits=3)


#Effects of type of training:

#computerized


COMPUTERIZZATOA <- rma.mv(y, v, mods = ~ TrComput, random = list(~ 1 | effectsizeID, ~
1 | studyID), tdist=TRUE, data=dataset)
summary(COMPUTERIZZATOA, digits=3)

dataset$TrNonComp<- 1- dataset$TrComput

NONCOMPUTERIZZATOA <- rma.mv(y, v, mods = ~ TrNonComp, random = list(~ 1 | effectsizeID, ~
                                                                      1 | studyID), tdist=TRUE, data=dataset)
summary(NONCOMPUTERIZZATOA, digits=3)




COMPUTERIZZATOB <- rma.mv(y, v, mods = ~ TrComput, random = list(~ 1 | effectsizeID, ~
                                                                  1 | studyID), tdist=TRUE, data=dataset2)
summary(COMPUTERIZZATOB, digits=3)
```

```
dataset2$TrNonComp<- 1- dataset2$TrComput

NONCOMPUTERIZZATOB <- rma.mv(y, v, mods = ~ TrNonComp, random = list(~ 1 | effectsizeID, ~
                                                                      1 | studyID), tdist=TRUE, data=dataset2)
summary(NONCOMPUTERIZZATOB, digits=3)



# invidividual vs. group

INDIVIDUALEA <- rma.mv(y, v, mods = ~ TrIndiv, random = list(~ 1 | effectsizeID, ~
1 | studyID), tdist=TRUE, data=dataset)
summary(INDIVIDUALEA, digits=3)

dataset$TrGruppo <- 1-dataset$TrIndiv
DIGRUPPOA <- rma.mv(y, v, mods = ~ TrGruppo, random = list(~ 1 | effectsizeID, ~
1 | studyID), tdist=TRUE, data=dataset)
summary(DIGRUPPOA, digits=3)

INDIVIDUALEB <- rma.mv(y, v, mods = ~ TrIndiv, random = list(~ 1 | effectsizeID, ~
                                                              1 | studyID), tdist=TRUE, data=dataset2)
summary(INDIVIDUALEB, digits=3)

dataset2$TrGruppo <- 1-dataset2$TrIndiv

DIGRUPPOB <- rma.mv(y, v, mods = ~ TrGruppo, random = list(~ 1 | effectsizeID, ~
                                                            1 | studyID), tdist=TRUE, data=dataset2)
summary(DIGRUPPOB, digits=3)


##########effects if number of sessions (we set a minimum of 10) and of duration of the training in minutes

NUMSESSA <- rma.mv(y, v, mods = ~ Nsess, random = list(~ 1 | effectsizeID, ~
                                                         1 | studyID), tdist=TRUE, data=dataset)
summary(NUMSESSA, digits=3)

NUMSESSB <- rma.mv(y, v, mods = ~ Nsess, random = list(~ 1 | effectsizeID, ~
1 | studyID), tdist=TRUE, data=dataset2)
summary(NUMSESSB, digits=3)

MINUTIA <- rma.mv(y, v, mods = ~ Minutes, random = list(~ 1 | effectsizeID, ~
                                                          1 | studyID), tdist=TRUE, data=dataset)
summary(MINUTIA, digits=5)

MINUTIB <- rma.mv(y, v, mods = ~ Minutes, random = list(~ 1 | effectsizeID, ~
1 | studyID), tdist=TRUE, data=dataset2)
summary(MINUTIB, digits=5)


############NEAR AND FAR TRANSFER
NEARA <- rma.mv(y, v, mods = ~ Near, random = list(~ 1 | effectsizeID, ~
                                                     1 | studyID), tdist=TRUE, data=dataset)
summary(NEARA, digits=3)

dataset$Far<-1-dataset$Near

FARA <- rma.mv(y, v, mods = ~ Far, random = list(~ 1 | effectsizeID, ~
                                                   1 | studyID), tdist=TRUE, data=dataset)
summary(FARA, digits=3)


NEARB <- rma.mv(y, v, mods = ~ Near, random = list(~ 1 | effectsizeID, ~
                                                        1 | studyID), tdist=TRUE, data=dataset2)
summary(NEARB, digits=3)

dataset2$Far<-1-dataset2$Near

FARB <- rma.mv(y, v, mods = ~ Far, random = list(~ 1 | effectsizeID, ~
                                                        1 | studyID), tdist=TRUE, data=dataset2)
summary(FARB, digits=3)



################ additional outcomes:


dataset<-dataset_complete
table(dataset$AddOutco)

min(dataset$v)
max(dataset$v)
mean(dataset$v)
sd(dataset$v)
table(dataset$v)
overall <- rma.mv(y, v, random = list(~ 1 | effectsizeID, ~ 1 | studyID), tdist=TRUE, data=dataset)

summary(overall, digits=3)

table(dataset$AddOutco)


ADDITIONAL <- rma.mv(y, v, mods = ~ AddOutco, random = list(~ 1 | effectsizeID, ~
                                                        1 | studyID), tdist=TRUE, data=dataset)
```
