# Supplementary figures and images for: Is Cognitive Training Effective for Improving Executive Functions in Preschoolers? A Systematic Review and Meta-Analysis
Source: Front Psychol. 2020 Jan 10;10:2812. doi: 10.3389/fpsyg.2019.02812 (PMC6965160; doi:10.3389/fpsyg.2019.02812)

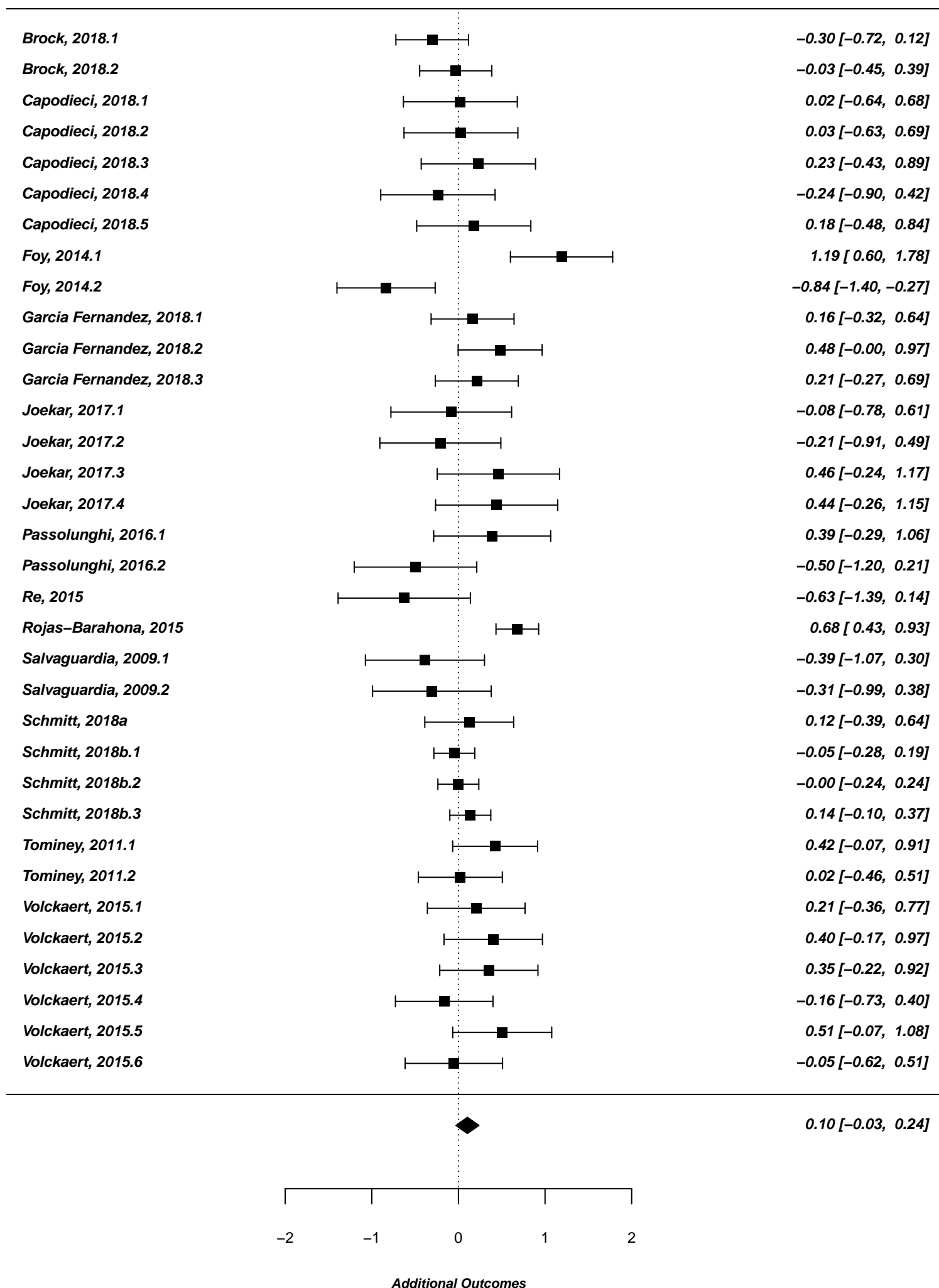

Supplement: Supplementary file 3 [file Table_3.PDF]

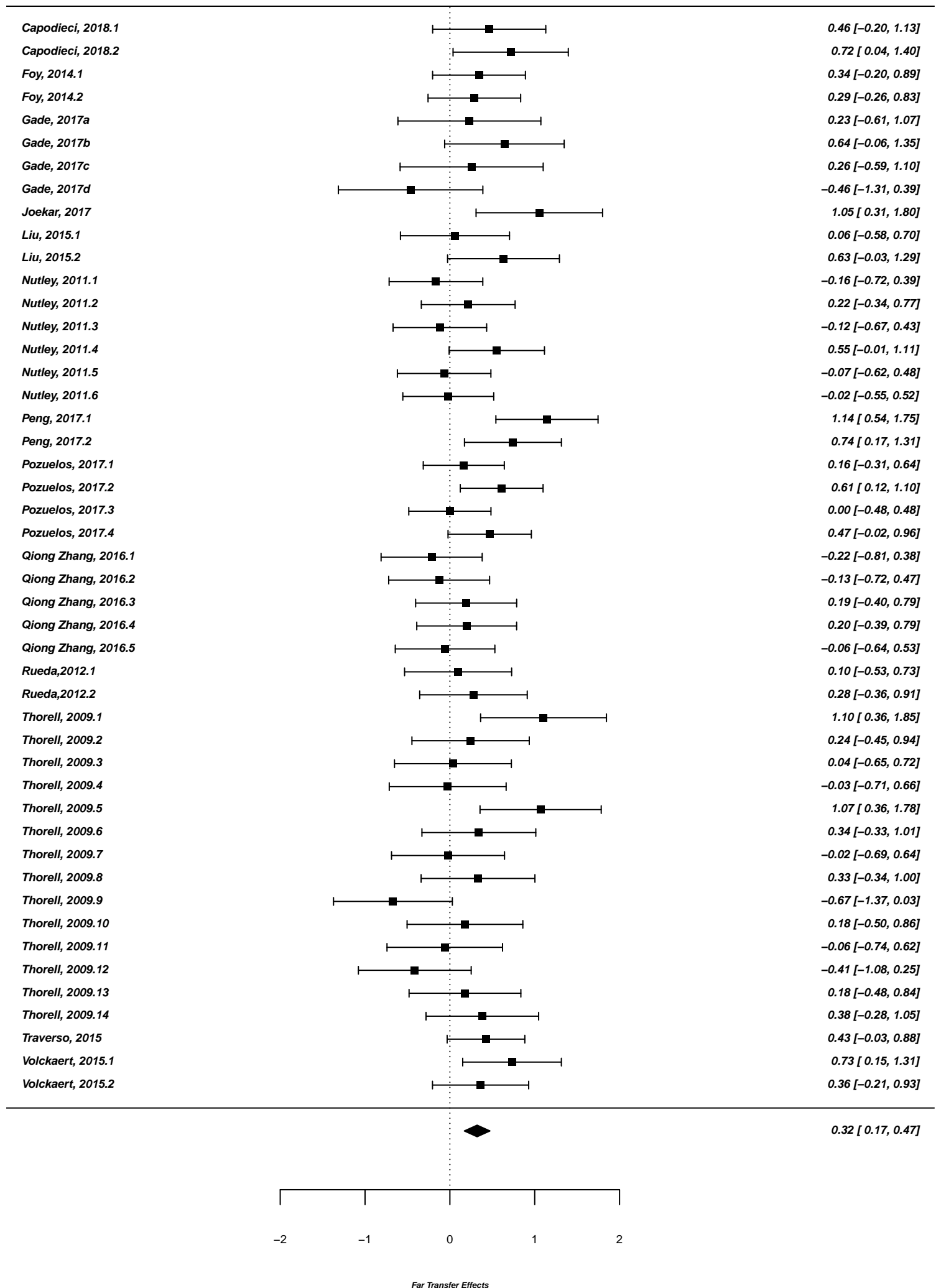

Supplement: Supplementary file 5 [file Table_5.PDF]

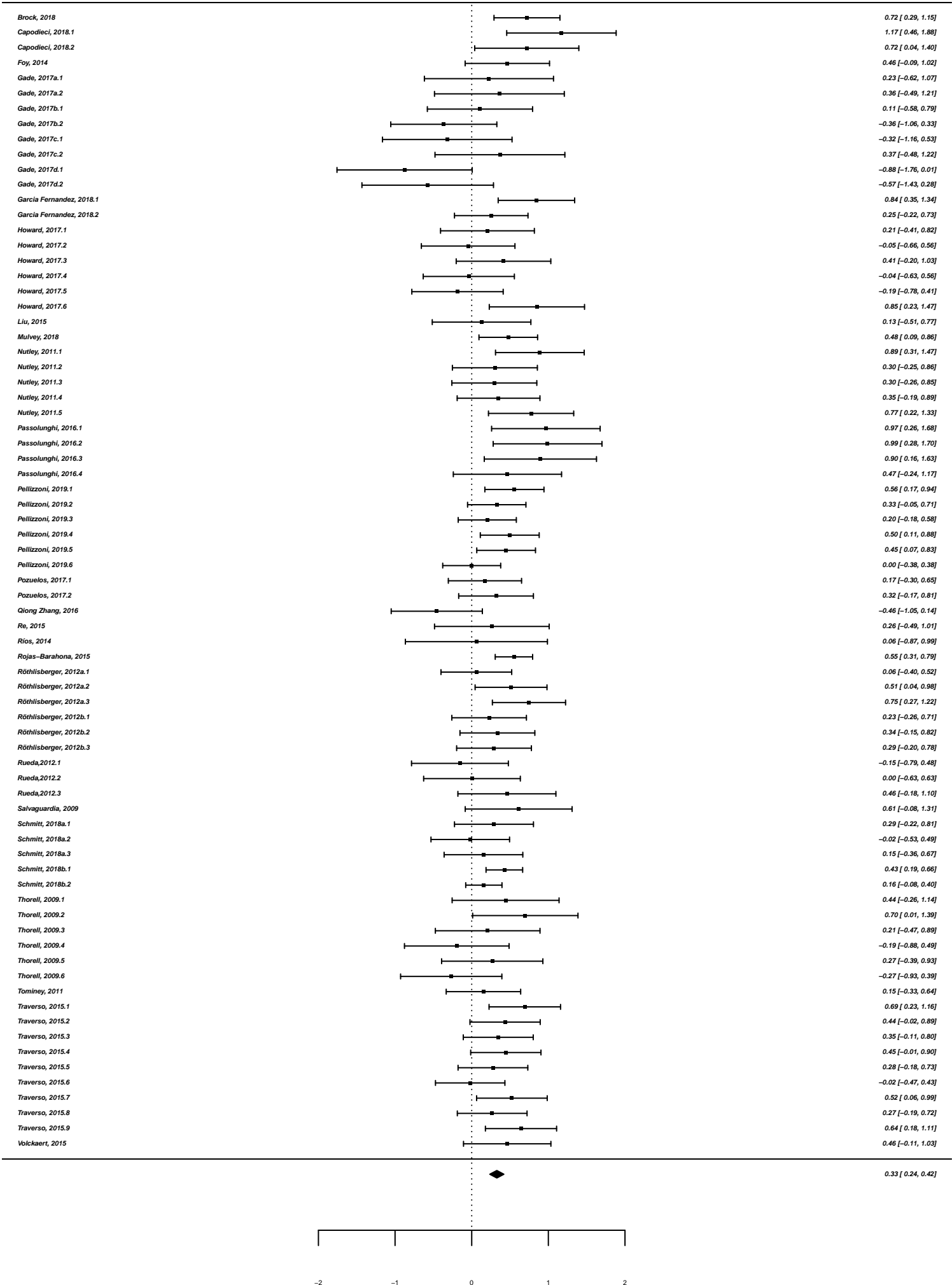

Supplement: Supplementary file 6 [file Table_6.PDF]
