# Supplementary material for: Is Cognitive Training Effective for Improving Executive Functions in Preschoolers? A Systematic Review and Meta-Analysis
Source: Front Psychol. 2020 Jan 10;10:2812. doi: 10.3389/fpsyg.2019.02812 (PMC6965160; doi:10.3389/fpsyg.2019.02812)
Supplement: Supplementary file 4 [file Table_4.PDF]

```
rm(list=ls())

# Setting the working directory;

setwd("C:/yourdirectory")

library(readxl)

DatiMeta <- read_excel("RawDataFromPapers.xlsx")
summary(DatiMeta)
str(DatiMeta)
head(DatiMeta)

DatiMeta$CorrectFactJ <- NA
DatiMeta$SD_pre <- NA
DatiMeta$y <- NA
DatiMeta$y2 <- NA
DatiMeta$EffectDirection2 <- NA



DatiMeta$CorrectFactJ <- (1 - 3 / (4*(DatiMeta$GSn + DatiMeta$GCn-2)-1))

#computation of g based on Morris (2008)

DatiMeta$SD_pre = sqrt ((DatiMeta$GSn-1) * DatiMeta$DSpreGS^2 + (DatiMeta$GCn-1) * DatiMeta$DSpreGC^2)  / sqrt(DatiMeta$GSn + DatiMeta$GCn-2)

DatiMeta$y = DatiMeta$CorrectFactJ *  ( (DatiMeta$MpostGS-DatiMeta$MpreGS)-(DatiMeta$MpostGC-DatiMeta$MpreGC) )  / DatiMeta$SD_pre
DatiMeta$y2 = DatiMeta$CorrectFactJ *  (DatiMeta$MchGS-DatiMeta$MchGC)  / DatiMeta$SD_pre #here in case there is the info about change but not the
means pre and post
DatiMeta$y[is.na(DatiMeta$y)]<-DatiMeta$y2[is.na(DatiMeta$y)]
DatiMeta$EffectDirection2<-DatiMeta$EffectDirection*(-2)+1
DatiMeta$y<-DatiMeta$y*DatiMeta$EffectDirection2 ##CORRECTION FOR THE DIRECTION OF THE EFFECT


#I manually correct the direction for the few studies for which we have only d and that have the effect wrongly coded
DatiMeta$cohens_d[DatiMeta$effectsizeID==37]<-0-DatiMeta$cohens_d[DatiMeta$effectsizeID==37]

#Here I compute g from d

DatiMeta$y2<-DatiMeta$cohens_d*DatiMeta$CorrectFactJ #here the case in which I only have Cohen's d
DatiMeta$y[is.na(DatiMeta$y)]<-DatiMeta$y2[is.na(DatiMeta$y)]

#here I also compute d, because I need it to compute the variability of g

DatiMeta$cohens_d=DatiMeta$y/DatiMeta$CorrectFactJ
```
