# Supplementary material for: Is Cognitive Training Effective for Improving Executive Functions in Preschoolers? A Systematic Review and Meta-Analysis
Source: Front Psychol. 2020 Jan 10;10:2812. doi: 10.3389/fpsyg.2019.02812 (PMC6965160; doi:10.3389/fpsyg.2019.02812)
Supplement: Supplementary file 7 [file Data_Sheet_1.PDF]

Metadata

The file data ‘FinalDataForMetaAnalysis.xslx’ contains all relevant information and the computed Hedge’s g and its variance, together with all relevant moderators.

Please note that the comma (,) is used as a separator for the decimals.

Variables included

| | |
|---|---|
| effectsizeID | Univocal identifier of each effect size |
| studyID | Univocal identifier of each study |
| comparison | Identifier of the effect size within the study |
| cite | Authors |
| year | Year of publication |
| grigia | 0 = published paper; 1 = unpublished |
| GCattivo | 0 = passive control group; 1 = active control group |
| GSn | Number of participants in the experimental group |
| GCn | Number of participants in the experimental group |
| GS_Meta | Mean age of participants in the experimental group |
| GSDSeta | Standard deviation of age of participants in experimental group |
| GCMeta | Mean age of participants in the control group |
| GCDSeta | Standard deviation of age of participants in control group |
| SvAt | Atypical Development (0=no, 1 = yes) |
| GrAt | Specific type of atypical development (ADHD or disadvantaged SES) |
| Abilita | Type of trained abiity |
| Task | Type of task |
| Effect Direction | 0 = higher values in the task indicate better performance; 1 = lower values indicate better performance |
| GrMult | Presence of more than 2 groups (1 = yes, 0 = no) |
| Contr | Comparison (for studies with more than 2 groups) |
| TrComput | Computerized Training (0= no, 1 = yes) |
| TrIndiv | Individual training (0=no, 1 =yes) |
| Nsess | Number of sessions |
| Minutes | Minutes of training (total) |
| AddOutco | Additional outcome. 0= measure of EF; 1 = measure different from EF |
| y | Hedges’ g (morris formula) |
| EffectDirection2 | Recoding of EffectDirection:<br>1 = higher values in the task indicate better performance; -1 = lower values indicate better performance |
| v | Variance of the effect |
| Near | Near transfer. 0 = far transfer; 1 = near transfer |
| Near_2 | Recoding of Near: 0 = far transfer; 1 = near transfer |
